# Mechanistic insights into the evolution of DUF26-containing proteins in land plants

**DOI:** 10.1038/s42003-019-0306-9

**Published:** 2019-02-08

**Authors:** Aleksia Vaattovaara, Benjamin Brandt, Sitaram Rajaraman, Omid Safronov, Andres Veidenberg, Markéta Luklová, Jaakko Kangasjärvi, Ari Löytynoja, Michael Hothorn, Jarkko Salojärvi, Michael Wrzaczek

**Affiliations:** 1 Organismal and Evolutionary Biology Research Programme, Viikki Plant Science Centre, VIPS, Faculty of Biological and Environmental Sciences, University of Helsinki, Viikinkaari 1 (POB65), FI-00014 Helsinki, Finland; 2 Structural Plant Biology Laboratory, Department of Botany and Plant Biology, University of Geneva, Geneva, Switzerland; 3 Institute of Biotechnology, University of Helsinki, Viikinkaari 5 (POB56), FI-00014 Helsinki, Finland; 4 School of Biological Sciences, Nanyang Technological University, 60 Nanyang Drive, Singapore, 637551 Singapore; 5 Present Address: Laboratory of Plant Molecular Biology, Institute of Biophysics AS CR, v.v.i. and CEITEC—Central European Institute of Technology, Mendel University in Brno, Zemědělská 1, 613 00 Brno, Czech Republic

## Abstract

Large protein families are a prominent feature of plant genomes and their size variation is a key element for adaptation. However, gene and genome duplications pose difficulties for functional characterization and translational research. Here we infer the evolutionary history of the DOMAIN OF UNKNOWN FUNCTION (DUF) 26-containing proteins. The DUF26 emerged in secreted proteins. Domain duplications and rearrangements led to the appearance of CYSTEINE-RICH RECEPTOR-LIKE PROTEIN KINASES (CRKs) and PLASMODESMATA-LOCALIZED PROTEINS (PDLPs). The DUF26 is land plant-specific but structural analyses of PDLP ectodomains revealed strong similarity to fungal lectins and thus may constitute a group of plant carbohydrate-binding proteins. CRKs expanded through tandem duplications and preferential retention of duplicates following whole genome duplications, whereas PDLPs evolved according to the dosage balance hypothesis. We propose that new gene families mainly expand through small-scale duplications, while fractionation and genetic drift after whole genome multiplications drive families towards dosage balance.

## Abstract (web summary)

Aleksia Vaattovaara et al. investigate the evolutionary history of a representative protein family, the DUF26-containing proteins, which is specific to land plants. They suggest that domain duplications and rearrangement led to the protein family’s two main subclasses.

## Introduction

Gene duplication and loss events constitute the main factor of gene family evolution^1^. Duplications occur by two major processes, whole-genome multiplications (WGM) and small-scale duplications (SSD), including tandem, segmental, and transposon-mediated duplications^2^. They appear to be distinct modes of expansion, since gene families evolving through WGMs rarely experience SSD events^3^. The division is also visible on the functional level, since genes duplicated in WGMs are enriched for signal transduction, transcriptional and developmental regulation as well as signal transduction functions, whereas SSDs occur preferentially on secondary metabolism and environmental response genes^3^. The prevailing hypothesis for the phenomenon is dosage balance; in regulatory networks and protein complexes the stoichiometric balance between different components needs to be preserved; thus, selection acts against losses following WGMs and against duplications in SSDs^4^. Families retained after WGMs are typically stable across different species whereas highly variable families evolve through SSDs^5^, suggesting high turnover rates. These results have been obtained by analyzing the extremes, top families displaying pure WGM retention or SSD characteristics^3^ while most gene families likely evolve in an intermediate manner.

Plants and other eukaryotes have developed a wide range of signal transduction mechanisms for controlling cellular functions and coordinating responses on cell, tissue, organ, and organismal level. Plants in particular encode large gene families of secreted proteins^6–8^ and proteins with extracellular domains to respond to environmental and developmental cues. However, the large numbers make it difficult to dissect conserved or specialized functions^4^, and therefore a detailed understanding of their evolution and duplication history in different plant lineages is needed. Signaling proteins with extracellular domains include receptor-like protein kinases (RLKs)^9,10^ and receptor-like proteins (RLPs)^11^. In RLKs, extracellular domains are involved in signal perception and protein–protein interactions^12^ while the intracellular kinase domain transduces signals to substrate proteins. RLKs are involved in essential mechanisms including stress responses, hormone signaling, cell wall monitoring, and plant development^12^. The large number of secreted proteins, RLKs, and RLPs in plants may reflect their sessile lifestyle and need for meticulous monitoring of signals from other cells, tissues, or the environment. Phylogenetic relations between different groups of RLKs and RLPs have been described^9,13–16^ but few have been physiologically and biochemically characterized^17^.

The Domain of Unknown Function 26 (DUF26; Gnk2 or stress-antifungal domain; PF01657)^18,19^ is an extracellular domain harboring a conserved cysteine motif (C-8X-C-2X-C) in its core. It is present in three types of plant proteins. The first class is CYSTEINE-RICH RECEPTOR-LIKE SECRETED PROTEINs (CRRSPs). The best characterized CRRSP is Gnk2 from *Gingko biloba* with single DUF26, which acts as mannose-binding lectin in vitro with antifungal activity^18,19^. Two maize CRRSPs have been shown to also bind mannose and participate in defence against a fungal pathogen^20^. The second class, CYSTEINE-RICH RECEPTOR-LIKE PROTEIN KINASES (CRKs), has a typical configuration of two DUF26 in the extracellular region and forms a large subgroup of RLKs in plants. CRKs participate in the control of stress responses and development in Arabidopsis and in rice^21–31^. The third class of DUF26 domain-containing proteins is the PLASMODESMATA-LOCALIZED PROTEINS (PDLPs). PDLPs contain two DUF26 domains in their extracellular region and a transmembrane helix, but lack a kinase domain. They associate with plasmodesmata and are involved in symplastic intercellular signaling^32^, pathogen response^33^, systemic signaling^34^, control of callose deposition^35^ and are targets for viral movement proteins^36^. However, the precise biochemical functions of plant DUF26-containing proteins remain unclear.

Tandem expansions drive the evolution of, for example, F-Box proteins^37^, and transcription factors^38^, but also RLKs^16^ and RLPs^11^. Diversification processes include sub-functionalization, where paralogs retain a subset of their ancestral functions, and neofunctionalization, where duplicated proteins acquire new functions^38^. CRKs and CRRSPs typically exist in clusters on plant chromosomes^24^, suggesting relatively recent tandem expansions. This makes DUF26-containing proteins a perfect dataset for sequence-based evolutionary investigation.

Here we carry out an in-depth analysis of the DUF26-containing proteins, a protein family involved in signaling, to explore the dynamics and effect of the different duplication mechanisms on overall gene family evolution. We combine phylogenetic analyses with experimental structural biology to gain insight into the evolution of DUF26-containing proteins. While sequence analysis indicates that the DUF26 domain is specific to land plants, the domain shows structural similarity to fungal carbohydrate-binding lectins. Our results suggest that DUF26-containing proteins constitute a group of carbohydrate-binding proteins in plants. CRKs and CRRSPs experienced both ancestral and recent lineage-specific tandem duplications. In contrast to the general pattern of gene families expanding by small-scale duplication events, these gene families experienced expansion also during or after WGMs. Our work illustrates that detailed understanding of the evolution of large protein families is a prerequisite for translating findings from model plants to different species and for dissecting conserved or specialized functions of proteins.

## Results

### DUF26-containing proteins have diverse domain compositions

**Fig. 1:**
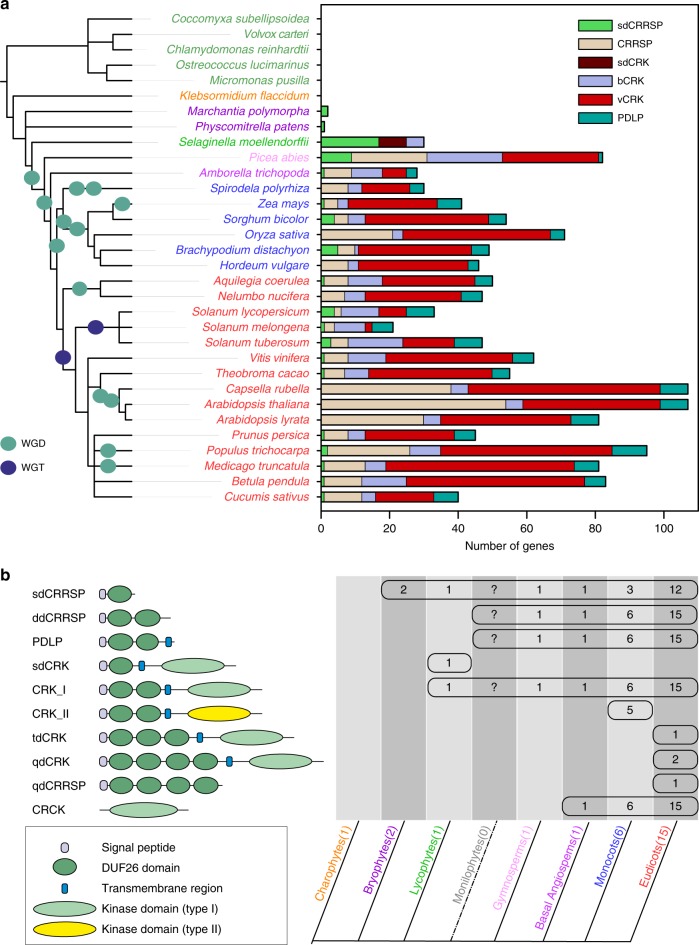
Overview and distribution of DUF26-containing genes in plants. **a** DUF26-containing genes are absent from algae and charophytes but present in land plants. *Marchantia polymorpha* and *Physcomitrella patens* genomes encode sdCRRSPs. *Selaginella moellendorffii* possesses sdCRRSPs, sdCRKs, and canonical CRKs. Seed plant (gymnosperm and angiosperm) genomes encode the whole set of DUF26-containing genes. CRKs were defined as basal group CRKs (bCRKs) or variable group CRKs (vCRKs) based on their phylogenetic positions. Whole-genome duplication (WGD) events are presented with green circle and whole-genome triplication (WGT) events with dark blue circle. Ferns were omitted from analyses due to lack of available genome assemblies. **b** Overview of different domain compositions of proteins containing DUF26 in different plant lineages. The number of representative species in the analyses is given in brackets after the name of the group. Numbers in the table present the number of species in each lineage in which the domain structure was found. Abbreviations sd (single domain), dd (double domain), td (triple domain), and qd (quadruple domain) refer to the number of the DUF26 domains

We selected 32 species with high-quality genome assemblies representing major plant lineages. After manual curation, de novo annotation, and exclusion of partial gene models and pseudogenes, we obtained 1409 high-quality gene models (Fig. 1a, Supplementary Figure 1, Supplementary Note 1, Supplementary Data 1). The PFAM protein domain database^39^ identifies DUF26 as specific to embryophytes. Accordingly, we identified no DUF26 or DUF26-like domains from genomes of algae, charophytes, diatoms, fungi, insects or vertebrates. DUF26-containing proteins are grouped into three categories: CRRSPs, PDLPs, and CRKs (Fig. 1b). CRRSPs consist of a signal peptide followed by one or more DUF26 domains, separated by a variable region. CRRSPs with a single DUF26 (sdCRRSPs) were identified from most land plants, including the liverwort (*Marchantia polymorpha)* and moss (*Physcomitrella patens)* lineages (Fig. 1). CRRSPs with two DUF26 domains (ddCRRSPs) were identified from vascular plants including the lycophyte *Selaginella moellendorffii* and represent the predominant type in vascular plants (Fig. 1). Rice as well as Brassicaceae display lineage-specific evolution with a large number of ddCRRSPs while sdCRRSPs are absent (Fig. 1a, Supplementary Figure 2).

CRKs contain a signal peptide, two DUF26 domains, and a transmembrane region followed by an intracellular protein kinase domain. Similar to ddCRRSPs, CRKs were identified from vascular plants but not from bryophytes (Fig. 1a). The CRKs likely originate from a fusion of sdCRRSPs with transmembrane region and kinase domain from LRR_clade_3 RLKs in the common ancestor of vascular plants^15^. The *Selaginella* genome uniquely encodes single DUF26 CRKs (sdCRKs; Fig. 1b) and only few CRKs from eudicots contain more than two DUF26 domains.

PDLPs were identified from all seed plants and are composed of a signal peptide, two DUF26, and a transmembrane region followed by short cytoplasmic extension. We also identified several angiosperm CRKs lacking signal peptide, extracellular domain, and transmembrane region. These are subsequently referred to as CYSTEINE-RICH RECEPTOR-LIKE CYTOPLASMIC KINASEs (CRCKs).

### Evolution of CRKs, PDLPs, and ddCRRSPs from small sdCRRSPs

**Fig. 2:**
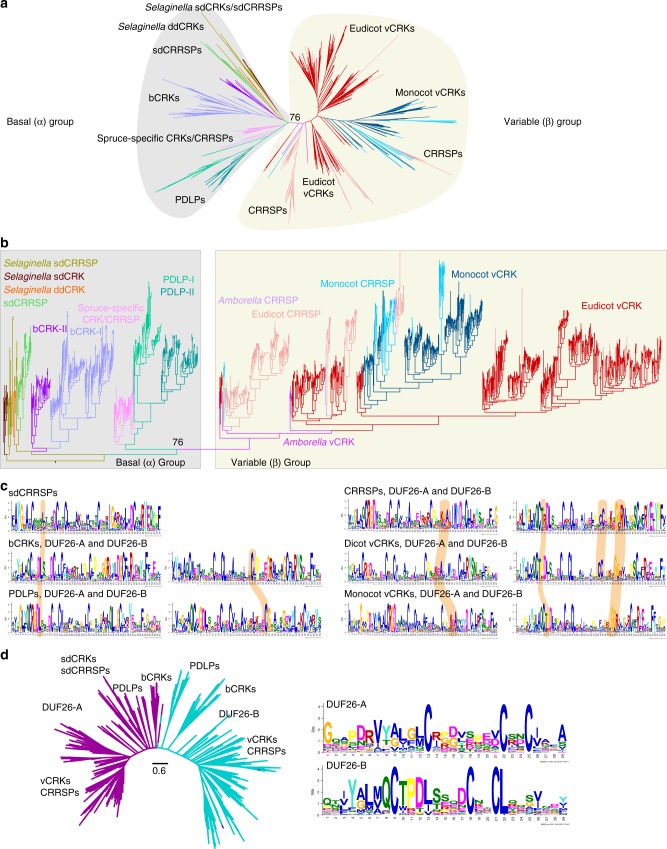
Phylogenetic tree of CRRSPs, CRKs, and PDLPs. **a** The phylogenetic tree was estimated with the maximum-likelihood method using all high-quality full-length DUF26-containing sequences from lycophytes onwards. CRCKs and concA-CRKs were excluded while GNK2 from *Gingko biloba* was included. Overall, DUF26-containing genes split into basal and variable groups. Detailed phylogenetic trees with bootstrap support (1000 replicates) and filtered sequence alignments are available at http://was.bi?id=IaroP (full tree), http://was.bi?id=wpEHGt (basal group separately), and http://was.bi?id=aIJe_D (variable group separately). **b** The same phylogenetic tree as in panel **a** rooted to ancestral sdCRRSPs and sdCRKs from *Selaginella moellendorffii* showing that the variable group branches out from the basal group. **c** The MEME figures present the conservation pattern of amino acid positions around the main cysteine motif within the DUF26 domains for sdCRRSPs, bCRKs, and PDLPs from the basal group and CRRSPs and vCRKs from the variable group. The features specific only to genes either in the basal group or in the variable group are highlighted. **d** The DUF26-A and DUF26-B domains are clearly separated in an unrooted phylogenetic tree containing DUF26 domain sequences. The MEME figures present differences in the conservation of the AA sequence surrounding the conserved cysteines in DUF26-A and DUF26-B

We investigated the evolution of CRRSPs, CRKs, and PDLPs by estimating phylogenetic trees (Supplementary Note 2). Overall, the tree of DUF26-containing proteins split into two distinct groups, basal group α and variable group β (Fig. 2a, b), where α is paraphyletic with respect to β. To increase the number of informative sites and obtain better resolution, we estimated separate phylogenetic trees for both groups (Supplementary Figure 2a, b). The subgroupings of the basal α- and variable β-groups remained conserved also in trees estimated for each subfamily of DUF26-containing proteins (Supplementary Figure 2c–e). Subsequently, we reconciled the gene trees with the species tree, and estimated ancestral gene contents and duplication/loss events for the subfamilies in 11 species (Supplementary Figure 3). To identify significant expansions we fitted birth–death rate models for DUF26-containing protein families and compared the rates against computationally derived gene families (orthogroups) for RLKs, all protein kinases, and plasmodesmal proteins^40^ using Badirate^41^. Finally, we assessed selective pressure by estimating amino acid conservation patterns around the main cysteine motif of the DUF26 domains for major subfamilies, and found conserved sites specific to α- or β-groups (Fig. 2, Supplementary Note 3).

The α-group is likely older, containing sequences from all vascular plants. Proteins in this group are conserved on sequence level and identification of putative orthologs from different species is frequently possible. Purifying selection, selective removal of (deleterious) variations, is likely the main force acting on this clade, as suggested by low *d*_*N*_*/d*_*S*_ values (one-rate model for whole groups: bCRK-I 0.184, bCRK-II 0.192, PDLPs 0.267, sdCRRSPs 0,162, CRCK 0.134; more flexible model with branch-specific *d*_*N*_*/d*_*S*_ within each group yielded similar results). The subgroups within the basal α-group evolved independently and their DUF26 domains share features distinguishing them from the β-group (Fig. 2c, Supplementary Note 3). Since the sdCRRSPs are located close to the root of the α-group (Fig. 2b) and form a monophyletic subclade at the root of the CRRSP tree (Supplementary Figure 2c), they are likely the ancestral type of DUF26 proteins in land plants. Furthermore, sdCRRSPs are present in various early diverging plant lineages such as the gymnosperm *Ginkgo biloba* (including Gnk2^18,19^) and the liverwort, *Marchantia polymorpha* (Supplementary Figure 2f). Turnover rates of sdCRRSPs do not differ from those of all gene families and show lineage-specific expansions in early diverging species (Supplementary Figure 3a).

**Fig. 3:**
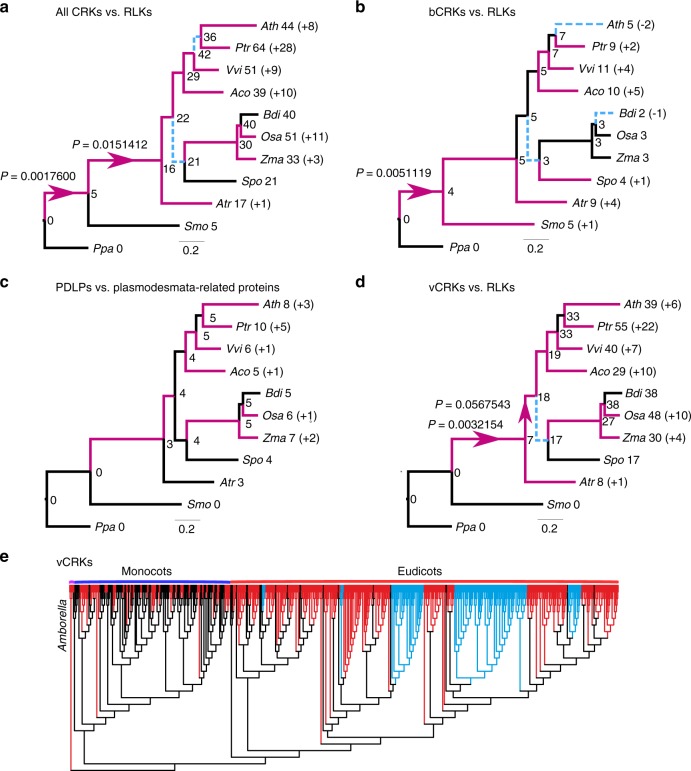
Comparison of evolutionary rates between gene families. Analyses were carried out with Badirate for 11 species (*Physcomitrella patens*, *Selaginella moellendorffii*, *Amborella trichopoda*, *Arabidopsis thaliana*, *Populus trichocarpa*, *Vitis vinifera*, *Aquilegia coerulea*, *Spirodela polyrhiza*, *Zea mays*, *Oryza sativa,* and *Brachypodium distachyon*). Neutral branches are reported as bold black lines; branches involving gene family expansion are reported as bold purple lines and branches with contraction as blue dashed lines. Branches with significant differences (false discovery rate adjusted *p* < 0.05) to birth–death rate model estimates are marked with arrows. Node labels present the ancestral gene family sizes estimated by Badirate. Tip labels contain species abbreviations and the change in numbers compared to the most recent ancestral node. **a** All CRKs compared to other receptor-like kinases (RLKs). **b** bCRKs compared to RLKs. **c** PDLPs compared to other plasmodesmata-related orthogroups. **d** vCRKs compared to RLKs. **e** Phylogenetic maximum-likelihood tree showing differences in lineage-specific expansions in monocot and dicot vCRKs following the split of *Amborella trichopoda*. Species-specific expansions (at least two genes from same species) are marked with red and clades including sequences from only Brassicaceae or Solanaceae are marked with blue

The placement of *Selaginella* sdCRKs to the root of the CRK phylogeny (Supplementary Figure 2d) and as sister to sdCRRSPs in the α-group (Fig. 2b) suggests an ancient origin. The DUF26 domain likely duplicated after fusing with transmembrane region and kinase domain, thus establishing the typical double DUF26 CRK configuration (Supplementary Figure 2d). Following duplication, the two DUF26 domains diverged into distinct, evolutionarily conserved, forms, DUF26-A and DUF26-B (Fig. 2d). All CRKs expanded significantly in the branches leading to lycophytes (*P* = 0.0017600) and to angiosperms (*P* = 0.0151412) compared to all RLKs (Fig. 3), and in the branch from lycophytes to angiosperms compared to all protein kinases (Supplementary Figure 4a).

A monophyletic group of CRKs with representatives from gymnosperms and angiosperms is located near the base of the CRK phylogeny (Supplementary Figure 2d) in the α-group (Figs 2a, b). This group, referred to as basal CRKs (bCRKs), likely represents the ancient CRKs in seed plants. Following the initial innovation in ancestral vascular plants, the group evolved at rates similar to orthogroups containing all protein kinases or all RLKs (Fig. 3b, Supplementary Figures 3b and 4b). The bCRKs split into two subgroups, bCRK-I and bCRK-II (Fig. 2b and Supplementary Figure 5), in gymnosperms and angiosperms, suggesting divergence in early seed plants. The larger bCRK-I subclade further divides into distinct branches with tandemly duplicated *Amborella* bCRKs at their roots (Supplementary Figures 5 and 6a, b) suggesting rapid differentiation following duplication in ancestral angiosperms^42^. The number of the bCRK-Is is conserved, excluding an expansion specific to Solanaceae, while the small bCRK-II subclade is absent from Brassicaceae.

PDLPs were only found in seed plants. PDLPs belong to the α-group (Fig. 2a, b) and represent the most conserved class of DUF26-containing genes. PDLPs do not display different expansion rates compared to plasmodesmata-related orthogroups^40^ (Fig. 3c). PDLPs split into two clades, PDLP-I and PDLP-II (Supplementary Figure 2e), which both contain *Amborella trichopoda* and eudicot and monocot PDLPs, suggesting divergence in ancestral angiosperms. PDLPs and ddCRRSPs originate from CRKs through loss of kinase domains and/or transmembrane regions. The loss can be a two-step process, as exemplified by an atypical PDLP from *Amborella trichopoda* located at the root of the main ddCRRSP clade (Supplementary Figure 7). PDLPs were possibly present already in ferns since database searches identified a partial gene model lacking a transmembrane region in *Marsilea quadrifolia*^43^ (see Methods) with similarity to PDLPs, placed at the root of a phylogenetic tree for PDLPs (Supplementary Figure 8).

In the α-group, a group of spruce-specific CRKs (spruce vCRKs) are more related to PDLPs than other CRKs (Fig. 2a, b). They form a distinct group between bCRKs and a large group of angiosperm variable CRKs (vCRKs; Fig. 2a, b, Supplementary Figures 2d and 3d). Angiosperm vCRKs form the β-group together with ddCRRSPs and atypical monocot sdCRRSPs (Fig. 2a, b). These CRRSPs likely evolved from vCRKs through loss of transmembrane regions and kinase domains and, in case of sdCRRSPs, also DUF26-B domains. The β-group is less conserved compared to the α-group and branches into two eudicot-specific and one monocot-specific group with a few *Amborella trichopoda* vCRKs at the root of the groups. Members of the β-group experienced several independent tandem expansions in different plant taxa (Figs 3d, e, Supplementary Figures 3d, 4c and 6c) and expanded during the diversification of monocots and dicots. CRRSPs in the β-group are not monophyletic, suggesting independent birth from partial duplications of vCRKs. Hence, expansion rates and extrapolation of ancestral gene counts for ddCRRSPs could not be reliably predicted. Lineage-specific expansions in the β-group make ortholog identification challenging.

### DUF26 is related to fungal lectins and assembles as tandems

High sequence divergence of DUF26 proteins and the lineage-specific expansions raise the question whether their overall structure is conserved. The consensus DUF26 domain as defined in PFAM comprises ~90–110 amino acids. Structural information is currently available for the sdCRRSP Gnk2^19^ but not for ddCRRSPs, CRKs, or PDLPs, proteins with a double DUF26 configuration. Mechanistic constraints restrict the evolution of protein structures, and therefore understanding structural conservation provides clues for protein function.

**Fig. 4:**
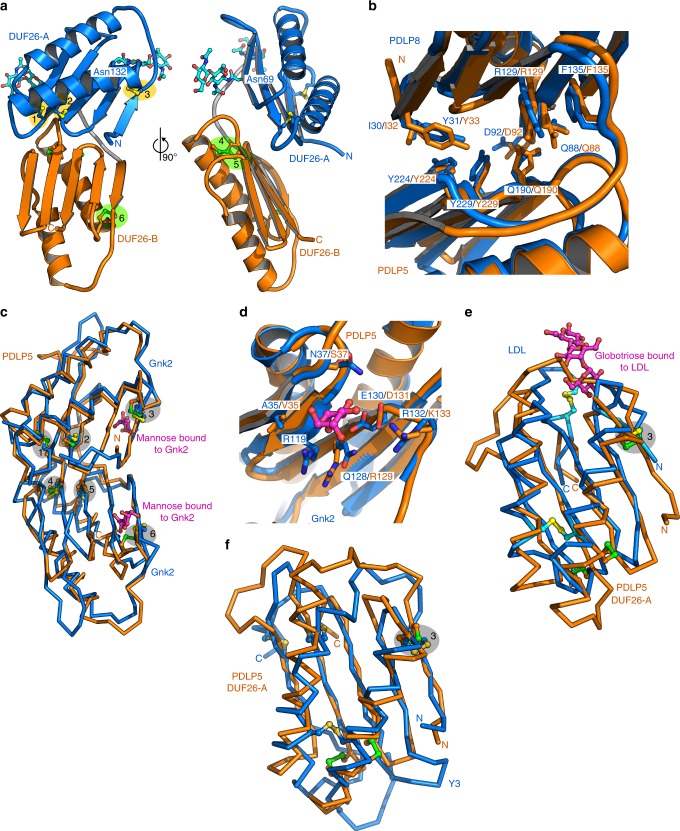
The crystals structures of the PDLP5 and PDLP8 ectodomains reveal a conserved tandem architecture of two lectin-like domains. **a** Overview of the PDLP5 ectodomain. The two DUF26 domains are shown as ribbon diagrams, colored in blue (DUF26-A) and orange (DUF26-B), respectively. N-glycans are located at Asn69 and Asn132 of DUF26-A and are depicted in bonds representation (in cyan). The DUF26-A and DUF26-B domains each contain three disulfide bridges labeled 1 (Cys89–Cys98), 2 (Cys101–Cys126), 3 (Cys36–Cys113), 4 (Cys191–Cys200), 5 (Cys203–Cys228), and 6 (Cys148–Cys215). **b** Close-up view of the DUF26-A–DUF26-B interface in PDLP5 (orange) and PDLP8 (blue), shown in bonds representation. **c** Superimposition of the Gnk2 extracellular DUF26 domain (PDB-ID 4XRE) with either PDLP5 DUF-26A (r.m.s.d. is ~1.4 Å comparing 100 aligned C_α_ atoms) or PDLP5 DUF26-B (r.m.s.d. is ~2.0 Å comparing 93 corresponding C_α_ atoms). Corresponding disulfide bridges shown in bonds representation (PDLP5 in green, Gnk2 in yellow) are highlighted in gray. Gnk2-bound mannose is shown in magenta (in bonds representation). **d** Close-up view of the residues involved in the binding of mannose of Gnk2 (bonds representation, in blue and magenta, respectively) and putative residues involved in ligand binding of PDLP5 DUF26-A (in orange). **e** The fungal LDL DUF26 domain (C_α_ trace in blue; PDB-ID 4NDV) and PDLP5 DUF26-A (in orange) superimposed with an r.m.s.d. of ~2.4 Å comparing 75 aligned C_α_ atoms). Disulfide bridges (LDL in yellow and PDLP5 in green; aligned disulfide bridges highlighted in gray) and the LDL bound globotriose (magenta) are shown in bonds representation. **f** C_α_ traces of the structural superimposition of the fungal Y3 protein (PDB-ID 5V6I) and PDLP5 DUF26-A (r.m.s.d. is ~2.6 Å comparing 78 corresponding C_α_ atoms). Disulfide bridges of Y3 (yellow) and PDLP5 DUF26-A (green) are shown alongside, one corresponding disulfide pair is highlighted in gray

We defined the structural relationship of tandem DUF26 domains by determining crystal structures of AtPDLP5 (residues 26–241) and AtPDLP8 (21–253) ectodomains to 1.25 and 1.95 Å resolution, respectively (Supplementary Table 1). Individual DUF26 domains feature two small α-helices folding on top of a central antiparallel β-sheet (Fig. 4). The PDLP5 DUF26-A domain is N-glycosylated at positions Asn69 and Asn132 in our crystals (Fig. 4a). The secondary structure elements of DUF26 are covalently linked by three disulfide bridges, formed by six conserved Cys residues, part of which belong to the C-8X-C-2X-C motif (Figs. 2d and 4a). We previously suggested that tandem DUF26-containing proteins could be involved in ROS or redox sensing^24,26^. To assess the functional roles of the invariant disulfide bridges in PDLPs, we mutated the partially solvent exposed PDLP5^Cys101^, PDLP5^Cys148^, and PDLP5^Cys191^ to alanine. While the wild-type PDLP5 ectodomain behaves as a monomer in solution (Supplementary Figure 9), the mutant protein tends to aggregate in our biochemical preparations (Supplementary Figure 9) and display reduced structural stability in thermofluor assays (Supplementary Figure 10). These experiments and crystallographic data (Fig. 4a) suggest that the conserved disulfide bonds in PDLPs and potentially in other DUF26-containing proteins are involved in structural stabilization rather than redox signaling.

The N-terminal DUF26-A (PDLP5 residues 30–132) and the C-terminal DUF26-B (residues 143–236) domains are connected by a structured loop (residues 133–142) and make extensive contacts with each other (Fig. 4a). The resulting ectodomain has a claw-like shape with the β-sheets of DUF26-A and B facing each other (Fig. 4a). The DUF26-A and B domains in PDLP5 and 8 closely align, with root mean square deviations (r.m.s.d.s) of 1.6 and 1.2 Å when comparing 89 corresponding C_α_ atoms, respectively (Supplementary Figure 11a). DUF26-A is more variable than DUF26-B on the sequence level (Fig. 2d). The DUF26-A and -B domains in PDLP5 and PDLP8 have 24% and 30% of their residues in common, most of which map to the hydrophobic core of the domain (including the six cysteine residues forming intra-molecular disulfide bonds) and to the DUF26-A–DUF26-B interface (Fig. 4b). This interface is formed by a line of aromatic and hydrophobic residues originating from the proximal face of the β-sheet in DUF26-A and -B (Fig. 4b, Supplementary Figure 12). Importantly, many of the interface residues are conserved among different PDLPs, but also among CRKs and ddCRRSPs (Supplementary Figure 12). Consistently, the ectodomains of PDLP5 and PDLP8 belonging to different phylogenetic clades (Supplementary Figure 8) closely align with an r.m.s.d. of ~1.6 Å when comparing 198 corresponding C_α_ atoms (Supplementary Figure 11b). These observations suggest that evolutionarily distant DUF26 tandem proteins likely share the conserved three-dimensional structure.

The physiological ligands for PDLPs are currently unknown. Therefore, we performed structural homology searches^44^ to obtain insights into the biochemical function of plant DUF26 domains (see Methods). Top hits include the single DUF26 domain protein Gnk2^19^. Despite moderate sequence similarity, the overall fold of Gnk2 and PDLP5 DUF26-A and B as well as disulfide-bond arrangement is conserved (Fig. 4c). Notably, Glu130 and Arg132 implicated in mannose binding in Gnk2 are replaced by Asp131 and Lys133 in the DUF-A of PDLP5, respectively (Fig. 4d). A similar pocket is found in the DUF-A domain of PDLP8, but not in the DUF-B domains of either PDLP5 or 8. Despite these structural homologies of Gnk2, PDLP5 DUF-A, and PDLP8 DUF-A, we could not detect binding of mannose to isolated PDLP5 ectodomain in vitro (Supplementary Figure 13a). We are also unable to detect any binding of other water-soluble cell wall-derived carbohydrates to the PDLP5 ectodomain (Supplementary Figure 13b). The PDLP5 DUF26 domains share strong structural homology also with two fungal lectins, the α-galactosyl-binding *Lyophyllum decastes* lectin (LDL)^45^ and a glycan-binding Y3 lectin from *Coprinus comatus*^46^. Both proteins closely align with the plant DUF26 domain, and share one of the three disulfide bridges (Fig. 4e–f). The surface areas involved in globotriose and glycan binding, respectively, are not conserved in PDLPs, but the structural similarity of plant DUF26 domains with different eukaryotic lectins could suggest a common evolutionary origin and a role as carbohydrate recognition modules^45^.

**Fig. 5:**
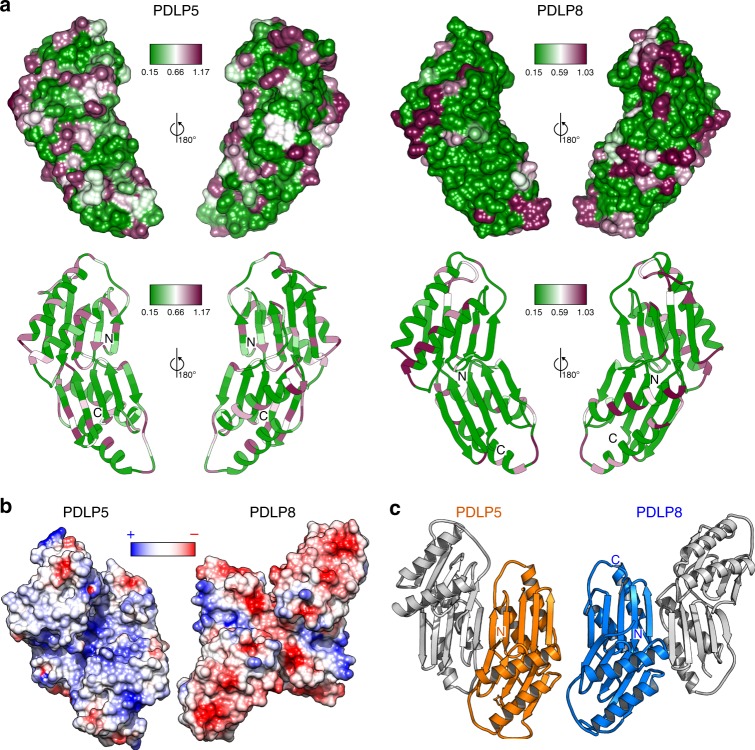
PDLP5 and PDLP8 may have drastically different oligomerisation modes; surface charge distributions and surface-exposed residues are not widely conserved. **a** The conservation of amino acid residues illustrated on the molecular surface of the PDLP5 or PDPL8 crystallization dimers, respectively. Sitewise *ω* (*d*_*N*_*/d*_*S*_) values, indicating the intensity and direction of selection on amino acid changing mutations, illustrated on the molecular surfaces and in ribbon diagrams of PDLP5 or PDPL8. The *ω*-values range from 0.15 (green) to slightly over 1.0 (magenta), reflecting conserved sites under purifying selection and sites evolving close to a neutral process, respectively. **b** Electrostatic potential mapped onto molecular surfaces of the putative PDLP5 and PDLP8, orientation as in **c** dimer, respectively. **c** Ribbon diagrams of PDLP5 (orange) and PDLP8 (blue) crystallographic dimers. In both dimers large, antiparallel β-sheets are formed, using different protein–protein interaction surfaces

We next explored potential binding sites in the two molecules by analyzing sitewise *ω* for orthologs of PDLP5 and PDLP8. Low *ω* values were observed in structural context, indicating conservation of residues buried inside the DUF26 domain, while variable residues (under more relaxed selection) appear on the surface of the structure (Fig. 5a). The variability of the PDLP5 and PDLP8 DUF26 domain surface may be central to their ability to interact with other proteins or ligands (Supplementary Figure 13c). Selection patterns may differ between young lineage-specific and evolutionarily conserved proteins. For PDLP5, high *ω* values on the surface could indicate fast evolution leading to sub- or neofunctionalization, as PDLP5 orthologs originate from the recent lineage-specific duplication in Brassicaceae. The different surface charge properties of related PDLPs from Arabidopsis (Fig. 5b) suggest that different PDLPs and other DUF26-containing proteins sense a diverse set of ligands. While the nature of these molecules is currently unknown, cell-wall-derived carbohydrates or small extracellular molecules represent candidate ligands. Notably, we observed typical lectin-dimers in PDLP5 and PDLP8 crystals, in which two lectin domains dimerized along an extended antiparallel β-sheet (Fig. 5c)^47^. In principle, this mode of dimerization could form an extended binding cleft for a carbohydrate polymer, and presents an attractive activation mechanism for PDLPs and CRKs, where a monomeric ground state forms ligand-induced oligomers, as previously seen with plant LysM-domain-containing carbohydrate receptors^48^.

### The CRK kinase domain is related to LRR and S-locus RLKs

Kinase domains transduce signals by phosphorylating substrate proteins. Typically, the kinase domain has been used to investigate phylogenetic relationships between RLKs^9,15,16^. The CRK kinase domain is similar to the kinase domain of S-locus lectin and LRR RLKs from LRR_clade_3^15^ (Supplementary Data 2). Based on catalytic motifs in the kinase domains^49^ most CRKs seem to be active kinases and in vitro activity of several CRKs has been experimentally confirmed^25,28,30^. Most CRKs belong to the RD type^50,51^ considered capable of auto-activation but few non-RD CRKs are present in plants^49^.

**Fig. 6:**
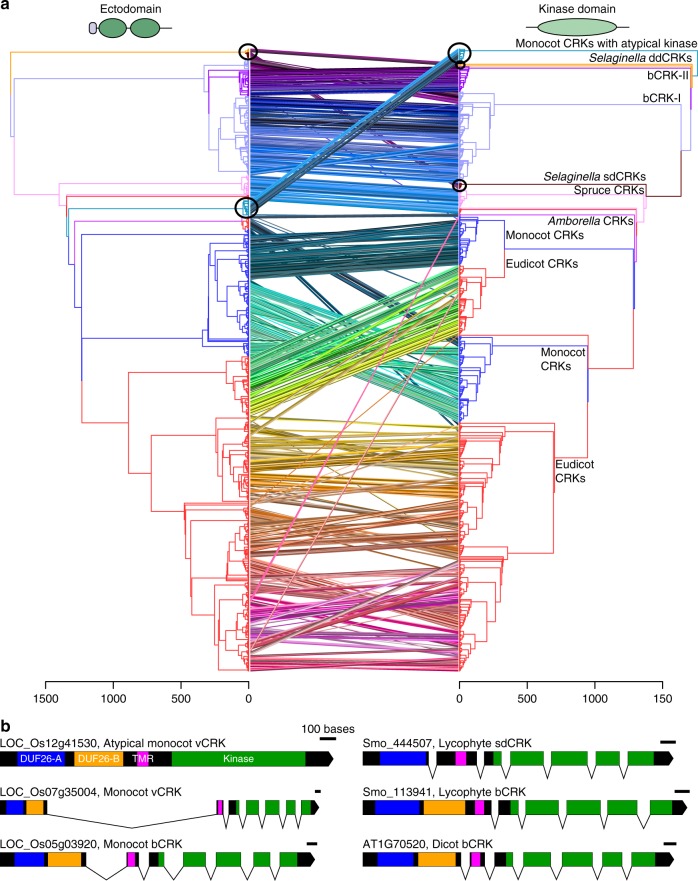
CRKs experienced domain rearrangements. **a** Comparison of phylogenetic trees based on ectodomain region and kinase domain of 880 CRKs. Phylogenetic maximum-likelihood trees are presented as tanglegram where the tree of the CRK ectodomain region is plotted against the tree of the kinase domain. The kinase tree is rooted to atypical monocot CRKs with a Concanavalin-A type kinase domain and the ectodomain tree is rooted to CRKs from *Selaginella moellendorffii*. The ectodomain tree was detangled based on the kinase domain tree. Lines connect the ectodomain and kinase domain belonging to same gene, and connection are drawn in different colors for better visibility. Juxtaposition of the trees shows rearrangements and domain swaps of ecto- and kinase domains. Black circles highlight the difference between the ectodomains and kinase domains of the *Selaginella* sdCRKs and ddCRKs and also the group of the atypical monocot CRKs which have exchanged the kinase domain. **b** The exon–intron structure of the CRKs. Usually CRKs contain seven exons: one encoding DUF26 domains, one encoding transmembrane region (TMR), and five exons encoding the kinase domain. In atypical monocot CRKs with exchanged kinase domain, whole gene is encoded by one or two exons. The scale bar for each gene represents 100 bases. Regions encoding the DUF26-A are colored with blue, the DUF26-B with orange, the transmembrane region (TMR) with pink, and the kinase domain with green

Analyzing ectodomains and kinase domains of CRKs separately suggests that *Selaginella* ddCRKs share an ancestor with bCRKs, while *Selaginella* sdCRKs share an ancestor with vCRKs (Fig. 6a). The separation of DUF26-A and DUF26-B (Fig. 2d) and the timing of those events does not reveal whether duplication of the DUF26 domain in the CRK extracellular region occurred more than once or whether functional constraints in the kinase domain led to the similarity of *Selaginella* sdCRKs and vCRKs. Juxtaposition of phylogenetic trees based on ectodomains and kinase domain suggests several exchanges of kinase or extracellular regions among CRKs during evolution (Fig. 6a). Most strikingly, a group of monocot-specific CRKs separates from other CRKs in a phylogenetic tree based on the kinase domain (Fig. 6a). Those CRKs have a different exon–intron structure (Fig. 6b, Supplementary Figure 1b) and a kinase domain with high similarity to concanavalin-A-like lectin protein kinase domains (Supplementary Data 2) altogether suggestive of chimeric gene formation following tandem duplication^52^. This kinase domain switch is specific to grasses (Poaceae) and has likely resulted in a different set of substrates. In addition, loss of ectodomains and transmembrane regions has established CRCKs at least three times; one group is specific to angiosperms (CRCK-I clade), one is specific to Brassicaceae and one only to *Arabidopsis thaliana*.

### Mixed-mode evolution of large gene families

**Fig. 7:**
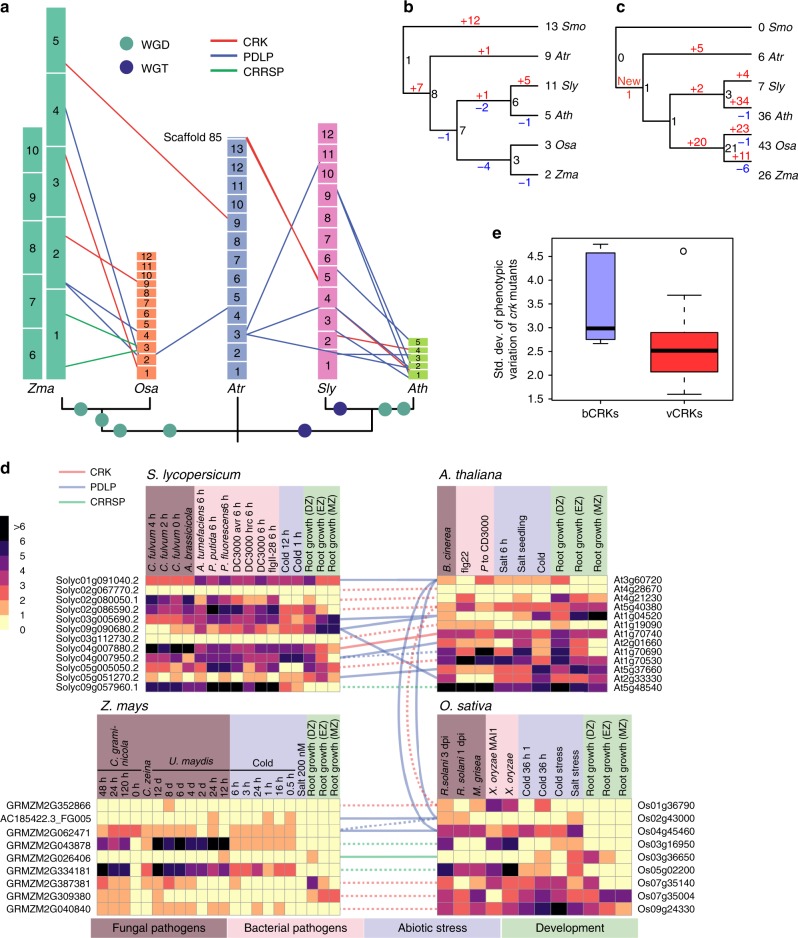
Identification of the modes of gene family evolution in DUF26-containing genes in *Arabidopsis thaliana*, tomato, rice, maize, and *Amborella trichopoda*. **a** Gene families that are preferentially retained after whole-genome multiplications (WGMs) are typically identified by synteny analysis. The figure illustrates syntenic regions containing DUF26 genes from *Amborella trichopoda* to monocots *Oryza sativa* and *Zea mays* and to eudicots *Solanum lycopersicum* and *Arabidopsis thaliana*. Within monocots and dicots, segments with at least five syntenic genes were included, whereas in comparisons to *Amborella* the minimum threshold was three syntenic genes. For *Amborella trichopoda* genomic locations of DUF26-containing genes are only known on chromosome/scaffold level based on physical mapping. **b**, **c** Gene families with a preferential retention pattern after WGMs show conserved gene counts over species. Phylogenetic tree of the five species (**a**) was used to reconcile the gene trees and estimate gene counts in ancestral nodes for **b** bCRKs and **c** vCRKs, using *Selaginella moellendorffii* as outgroup. The gains are highlighted with red and losses with blue. **d** Gene families with preferential retention pattern should have many orthologs. Heatmaps of the normalized transcriptional expression counts (Transcript per million [TPM]) of candidate DUF26 orthologs from four of the species: *Solanum lycopersicum*, *Arabidopsis thaliana*, *Zea mays*, and *Oryza sativa*. Coloring in heatmaps is proportional to log_2_ (TPM) value that represents the gene expression level. The corresponding log_2_ (TPM) value is displayed next to the color key. The rows represent gene models and the columns show the experiments, collected from publicly available Sequence Read Archive (SRA) database. SRA accessions are annotated to relevant stress conditions (descriptions are presented in Supplementary Data 4). Solid lines connect putative orthologs based on evidence from phylogenetic and synteny analyses; dashed lines connect putative orthologs based on evidence from either phylogenetic or synteny analyses. **e** Gene families evolving under dosage balance are predicted to demonstrate a high phenotypic effect in their knockouts. This can be seen by reanalysis of phenotype data from Bourdais et al.^24^; the bCRK T-DNA insertion mutants display a larger standard deviation (*Y*-axis) over different phenotyping experiments than vCRK mutants

For detailed analyses of gene family dynamics we analyzed the synteny, conservation of gene order between species, and tandem duplications in *Amborella trichopoda*, tomato (*Solanum lycopersicum*), Arabidopsis, rice, and maize *(Zea mays*; Fig. 7, Supplementary Figure 7), and estimated the timing of duplication events by reconciliation of gene trees with species trees (Supplementary Figure 14a).

Within the rapidly diverging β-group the vCRKs show large lineage-specific expansions. The ancestral origins for monocot and eudicot vCRKs differ, and neither synteny nor orthology can be identified (Fig. 7c, Supplementary Figure 14c). This suggests that this subfamily has a high birthrate and expands rapidly by tandem duplications. Additionally, many tandems are lost or fractionated after WGMs. Similarly, CRRSPs demonstrate little synteny between species (Fig. 7a, Supplementary Figure 14d), and CRRSPs in rice and *Arabidopsis* experienced lineage-specific tandem duplications (Supplementary Figure 14d). In Brassicaceae this expansion can be traced to *Amborella* CRRSP (*AtrCRRSP2*), suggesting a tandem mode of expansion.

Tandem duplications evolve through unequal crossover or homologous recombination events^53^. Unequal crossover produces copy number variation, whereas homologous recombination such as gene conversion plays a role in concerted evolution, which can maintain the similarity between gene copies over long periods^54^. Gene conversion depends on genomic distance as well as sequence homology. Accordingly, we observed several events among the lineage-specific tandem vCRK expansions (Supplementary Data 3), whereas for bCRKs gene conversion was only observed in the tandem expansion in *Amborella*. Thus, gene conversion is important for maintaining the similarity between recent tandem duplicates but conversion events become rare as sequences diverge over time.

The CRCK-I genes are present in most genomes as single copy genes within conserved syntenic genome segments, suggesting that duplicates from WGMs were lost during genome fractionation (Fig. 7a). Evolution follows a model where maintenance of a single copy is critical for the organism. A hallmark of gene families evolving under dosage balance is that their overall numbers should be conserved between species with similar WGM histories. In the species tree (Fig. 7a), most branches experienced one or two WGMs. Despite these events, the number of bCRKs is well conserved in angiosperms (Figs. 3b and 7b, Supplementary Figures 3b and 7b). However, in *Amborella trichopoda* five bCRK genes appear in tandem which form the roots of the respective orthologs (Supplementary Figure 6b), indicating an ancestral SSD origin still present in *Amborella*. The duplicate region experienced fractionation during evolution leading to Brassicaceae and Solanaceae lineages, resulting in scattered bCRK-I orthologs with little conserved synteny, whereas in grasses the tandem duplicate region was lost. This indicates rapid pseudogenization of the duplicated tandem blocks after WGMs, with, except for Solanaceae, no recent tandem expansions. This suggests that a gene family initially existing as a tandem duplicate may have shifted towards dosage balance evolution. Dosage balance is observed in another subfamily, PDLPs; they appear in genomic regions where synteny is conserved within eudicots and monocots (Fig. 7a), and no SSD events can be detected.

A prediction for gene families evolving under dosage balance is that retained duplicates should exhibit less functional divergence than other duplicates^3^. We explored functional conservation by analyzing publicly available gene expression data on stress treatments (Fig. 7d, Supplementary Data 4, Supplementary Figure 15). In agreement with previous studies^24,26,31,55^, pathogen treatments have the biggest impact on transcript abundance of DUF26-containing genes, in particular CRKs and CRRSPs (Supplementary Figure 15). Analysis of gene expression data suggest extensive lineage-specific functional diversification. This is visible in the correlation rank between putative orthologs; in many cases higher correlation is found with DUF26-containing genes that have less sequence similarity, indicating that closely related genes experienced sub- or neofunctionalization following duplication^56,57^.

Despite rearrangements and lineage-specific expansions the data provide support for seven putative orthologs, including three PDLP and three CRRSP relationships (Fig. 7d; Supplementary Data 4). Even though the synteny of bCRKs (and PDLPs) is more conserved compared to CRRSPs, bCRKs demonstrate varying responses to stimuli, whereas in CRRSPs synteny is associated with similar functions.

The second prediction from the dosage balance model is that, since the proteins encoded by the genes are highly connected and thus interact with many other proteins, disturbances in dosage balance should have large effects on an organism’s phenotype^58^. Reanalysis of phenotyping data *crk* mutants^24^ confirms that bCRKs indeed demonstrate a larger variance in phenotypes than vCRKs (*p* = 0.03; Wilcox test; Fig. 7e). Altogether the analysis suggests that PDLPs and bCRKs are evolving according to the dosage balance model, whereas the vCRKs and CRRSPs evolve by SSD mechanisms.

## Discussion

Compared to animal genomes, plant genomes encode a large number of large gene families^59^. In particular, signal transduction components including transcription factors, protein kinases, and phosphatases have experienced drastic expansions in plants^59^. This might reflect adaptation to a sessile lifestyle but also different signaling strategies on the cellular level. The large, in part lineage-specific, expansions and domain rearrangements hamper the identification of orthologous proteins in different plant species. Here we used the DUF26-containing proteins to study the evolution of a large plant protein family with heterogeneous domain architecture and drastic lineage-specific expansions. We identified 1409 high-quality gene models representing CRRSPs, CRKs, and PDLPs from major plant lineages. The sdCRRSPs are the ancestral type of DUF26-containing proteins. CRKs originate from a fusion of CRRSPs with transmembrane region and kinase domain of LRR_clade_3 RLKs^15^ in the lineage leading to lycophytes. PDLPs and ddCRRSPs emerged subsequently through loss of kinase and transmembrane domains. Our results reveal an ancient split into two distinct groups. The α-group is conserved in size and sequence throughout embryophytes. This facilitates identification of orthologs and extrapolation of functional information from model plants to crops. The β-group evolved before the split of monocots and eudicots and contains CRKs and CRRSPs that expanded through WGMs followed by lineage-specific tandem duplications. Domain rearrangements in the β-clade led to secondary groups of ddCRRSPs and sdCRRSPs while the recruitment of a different kinase domain in grasses suggests re-routing of signaling pathways. Domain exchanges in DUF26-containing proteins highlight the importance of comparing phylogenies inferred from full-length sequences with phylogenies inferred from individual domains. It is likely that members of the β-group have been subject to sub- and neofunctionalization, which is a challenge for functional analyses. WGMs have been associated with periods of environmental upheaval and increasing biological complexity^2,60^. Accordingly, the evolution and radiation of DUF26-containing proteins with different domain structures co-occur with the appearance of new physiological characteristics and the adaptation to new habitats and lifestyles (Fig. 1b).

Sequence analysis suggested that DUF26 proteins could be specific to embryophytes. Crystallographic analysis of PDLP ectodomains reveals that the structure of their DUF26 domains closely matches the fold of the evolutionarily distant sdCRRSP Gnk-2. PDLPs contain two DUF26 domains and the structure of Gnk-2 is more similar to DUF26-A but despite the structural similarity the mannose-binding function of Gnk-2 is not conserved. Intriguingly, plant DUF26 domains share strong structural similarity with fungal carbohydrate-binding modules but the tandem arrangement of two lectin-like DUF26 domains appears to be plant-specific. Rapid sequence divergence^61^ is a limiting factor in detection of homology at the amino acid sequence level, seen e.g. in the marked differences between DUF26 from bryophytes and those from other plants. This may obscure identification of ancestral proto-DUF26 domains in charophytes and algae. Our work suggests that different tandem DUF26 domains likely recognize diverse sets of ligands which still remain to be discovered. Similar to plant malectin receptors^62^, DUF26 domains may have evolved novel or additional functions which might include mediation of protein–protein interactions at the cell surface^20,35^. The strong structural similarity between DUF26 domains and fungal lectins suggests either a common origin or convergent evolution. DUF26 proteins represent carbohydrate-binding domains in plants and identification of ligands for different DUF26 domains will provide insights into perception of cell wall status or environmental signals. However, the large number of carbohydrates and related compounds in the plant cell wall may pose a challenge for this process.

**Fig. 8:**
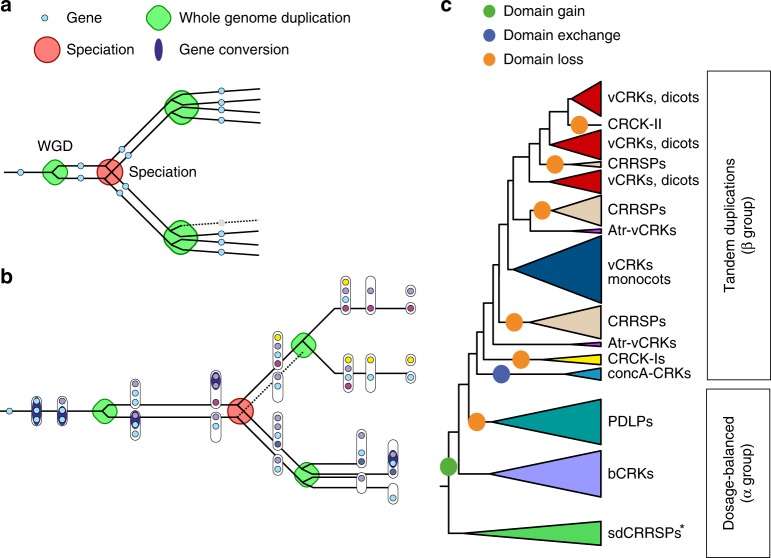
Model of mixed-type gene family evolution. Gene families evolve mainly through whole-genome multiplications (WGM) and small-scale duplications (SSD). Genes related to environmental responses and secondary metabolism experience SSDs, whereas highly connected genes associated with transcriptional and developmental regulation or signal transduction functions are preferentially retained after WGMs. **a** Prevailing hypothesis for the retention pattern is dosage balance; for highly connected genes the stoichiometric balance needs to be maintained, and therefore selection acts against gene losses after WGMs and against duplications by SSDs. **b** Gene family evolving through tandem duplications (**b**; evolution before the speciation node) has a high birthrate and therefore the number of duplicates between species can vary. After duplications the homogeneity of the duplicates may be maintained through gene conversion events occurring mainly within near-by homologous sequences. Over time, sequences eventually diverge by drift and selection. Our data suggest that tandemly expanding gene families may evolve into a dosage balance mode as a result of WGMs (**b**; evolution after speciation node). Following WGMs, duplicated tandems may experience extensive fractionation due to drift and selection by dosage fragmenting the tandem structure. At the same time, the connectivity of the gene family has been accumulating through sub- and neofunctionalization, increasing pressure for retention of genes. These phenomena together may result into a dosage balance model of evolution (top branch after speciation node). This does not necessarily occur across all WGM events and depends on the tandem duplication rate, as was observed for *bCRKs* in Solanaceae (bottom branch), where single copies and a later tandem expansion exist in the genome. Different subfamilies can be in different states of this process. **c** CRRSPs and PDLPs follow dosage balance mode after the paleohexaploid event, whereas *bCRKs* have assumed the mode in later WGM events. The overall numbers of the *bCRKs* are preserved but identification of orthologs between species that have experienced independent WGMs is difficult, suggesting recent convergent functionality of the members. Gene families expanding through tandem duplications such as vCRKs and CRRSPs have high birthrate and demonstrate several lineage-specific expansions. Asterisk indicates loss of sdCRRSPs in Brassicaceae and rice

From the analyses, an overall evolutionary model emerges (Fig. 8). After introduction to the genome, gene families may initially expand through tandem duplications and experience relaxed selection^63^. This is supported by the fact that the tandem genes function in processes requiring fast adaptation such as adaptation to environment, pathogen responses, and secondary metabolism^2,64^. Furthermore, tandem duplicates show high variation across species and have high *d*_*N*_*/d*_*S*_ rates^5^. In tandems, the main evolutionary forces are unequal crossover and concerted evolution through gene conversion, but over time genes evolve to gain specific functions. This process may be interrupted by WGM events. Since the tandem genes are not evolving under dosage balance, there is no compensatory drift^65^. Thus, drift and selection by dosage eventually drives some duplicates towards fixation while others turn into pseudogenes. Assuming that the elements driving tandem duplications are still present after fractionation, the remaining duplicates may in turn expand. In case of a tandem where all genes have established a unique functional role in the system, drift may drive duplicated tandems into scattered orthologs. These orthologs may assume a fixed syntenic position in the genome and a switch to a dosage balance mode of evolution results. The evolutionary mode of gene families depends on the balance between death rate after WGMs and birthrate of tandem duplications.

Our study of DUF26-containing proteins demonstrates the challenges in analyses of large protein families and the power of combining evolutionary and structural approaches. Our analysis provides a model for future studies of similarly large protein families and facilitates detailed biochemical and physiological investigation of the mechanistic functions of CRKs, PDLPs, and CRRSPs in different plant species.

## Methods

### Gene identification and annotation

Altogether 32 plant and algae genomes (Supplementary Data 1) covering the major plant lineages were selected for analyses. For 27 species protein annotations (primary transcripts) and genome sequence data were retrieved from Phytozome^66^ and Barley (*Hordeum vulgare*) from Gramene (http://www.gramene.org) with the latest names for gene models from IPK server (http://webblast.ipk-gatersleben.de/barley_ibsc/)^67^. Silver birch (*Betula pendula*) was sequenced at the University of Helsinki^56^. Eggplant (*Solanum melongena*) data were retrieved from Eggplant Genome DataBase (http://eggplant.kazusa.or.jp/). *Klebsormidium flaccium* and Sacred lotus (*Nelumbo nucifera*) genome data were from NCBI (https://www.ncbi.nlm.nih.gov). Additionally the FungiDB^68^ (www.fungidb.org), InsectBase^69^ (http://www.insect-genome.com) human (*Homo sapiens*), chicken (*Gallus gallus*), and zebrafish (*Danio rerio*) genomes were screened for DUF26. Detailed information of the genome versions and references are given in the Supplementary Table 2.

HMMER (version 3.1b2) search^70^ for PFAM domain with ID PF01657 (stress-antifungal domain) was carried out among AA sequences representing gene models from different species^71^. Genome sequences were checked with Wise2 (version 2.4.1) software^72,73^. All gene models found with HMMER were manually curated, and new genes found with Wise2 were manually annotated using Fgenesh+^74^. Birch (*Betula pendula*)^56^ and Sacred lotus (*Nelumbo nucifera*) were fully manually annotated as they did not have gene models a priori. High rates of manual annotation and curation were needed for *Selaginella moellendorffii*, grapevine (*Vitis vinifera;* version Genoscope.12X^75^) and potato (*Solanum tuberosum*). Sequences from each species were further checked by carrying out a multiple sequence alignment and phylogenetic tree estimation with PASTA^76^. Partial gene models were identified by checking sequences individually. Genes were defined as pseudogenes if the genomic sequence was available but no full domain structure could be predicted. In cases where the prediction problem was caused by the length of the contig or a gap in the genome sequence the gene model was marked as partial. Pseudogenes and partial gene models were not included in the subsequent analyses.

For domain analyses and phylogenetic trees containing only domain sequences, the domain borders were defined with HMMER using the PFAM domain PF01657 for DUF26 and PF07714 for the kinase domain from curated dataset. The ectodomain region was defined to end at the border of the transmembrane region in the PDLPs and CRKs. The partial PDLP from *Marsilea quandrifolia* was identified by using pBLAST search against sequences in the NCBI database.

### Phylogenetic trees

Only full gene models were used to infer phylogenetic trees. Sequence quality in alignments was checked using Guidance (version 2.01) and alignments were built using the MAFFT option^77^. Sequences with low-quality score were removed from datasets and alignments were built again with PASTA. For phylogenetic trees, alignments were filtered in Wasabi^78^ to remove residues with less than 10% coverage. Filtering was required due to the high sequence diversity (on less conserved regions) resulting in a high number of gaps in multiple sequence alignments. Maximum likelihood (ML) phylogenetic trees were inferred for filtered and also unfiltered data using RAxML (version 8.1.3)^79^.

ML phylogenetic trees were bootstrapped using RAxML (version 8.1.3) for 1000 bootstrap replicates. For phylogenetic trees containing full-length sequences with all domain structures bootstrapping was also carried out with partitioning (both DUF26 and kinase domains defined separately). The PROTGAMMAJTT model was used in phylogenetic analyses using RAxML. Model selection was based on a Perl script for identifying the optimal protein substitution model (available in RAxML webpage, provided by Alexandros Stamatakis). Bootstrapped trees are available on Wasabi^78^ (Supplementary Table 3). Comparison on phylogenetic trees based on CRK ectodomain and kinase domain regions was visualized in R using the dendextend package.

### Exon–intron structure

The number of exons for all genes was estimated using Scipio (version 1.4.1)^80^ using default parameters (minimum identity of 90% and coverage of 60%). It internally uses BLAT to perform the initial alignment of the protein sequences against the genome followed by refinement of hits to determine the exact splicing borders and to obtain the final gene structure. The number of exons per gene was extracted from the final result.

### Orthogroup generation

Eleven representative species from different clades (*Arabidopsis thaliana*, *Amborella trichopoda*, *Oryza sativa*, *Zea mays*, *Vitis vinifera*, *Populus trichocarpa*, *Aquilegia coerulea*, *Brachypodium distachyon*, *Physcomitrella patens*, *Selaginella moellendorffii,* and *Spirodela polyrhiza*) were chosen to study the evolution of the DUF26-containing proteins. Primary protein sequences of these 11 species were downloaded from Phytozome (version 11.0). An all-against-all BLAST was run for all the protein sequences followed by generation of orthogroups using the software OrthoMCL (version 2.0.9)^81^ with an inflation parameter of 1.5 for the clustering phase. Clustering yielded 34,535 orthogroups.

### Species tree generation

Orthogroups containing one representative protein for each of the 11 species were chosen to generate the species tree. Multiple sequence alignment was carried out on the single copy orthogroups using PRANK^82^ and the output was used to infer a species tree using RAxML^79^.

### Evolutionary rate and ancestral size estimation

The evolutionary rate and ancestral size of the orthogroups were modeled using Badirate software (version 1.35)^41^. The species tree and orthogroups generated from the previous steps were used as input for Badirate. The BDI (Birth, Death, Innovation) rate model was used. The Free Rates (FR) branch model was chosen which would assume every branch of the species tree to have its own turnover rates. Turnover rates of orthogroups were estimated using the ML fitting. Orthogroups were defined as protein kinases if they included sequences with PFAM domain PF00069. Orthogroups containing RLKs were defined based on known *Arabidopsis* RLKs^15^. Plasmodesmata-related orthogroups were defined based on *Arabidopsis thaliana* genes related to plasmodesmata^40^.

### Nucleotide coding sequence extraction for PAML

The GFF file output from Scipio^80^ was pre-processed by an in-house script and processed with the gff3 module of the GenomeTools (version 1.5.4)^83^ software. The final GFF file along with the corresponding species genome in fasta formatted file was passed as an input to the extractfeat module of the GenomeTools software to extract the final nucleotide coding sequences.

### PAML analyses

We estimated *d*_*N*_*/d*_*S*_ ratios (ratio of non-synonymous and synonymous sites, *ω*) for conserved clades (bCRK-I, bCRK-II, CRCKs (orthologs of AtCRK43), PDLPs, and sdCRRSPs) from 11 species (*Arabidopsis thaliana*, *Amborella trichopoda*, *Oryza sativa*, *Zea Mays*, *Vitis vinifera*, *Populus trichocarpa*, *Aquilegia coerulea*, *Brachypodium distachyon*, *Physcomitrella patens*, *Selaginella moellendorffii*, and *Spirodela polyrhiza*) by using the codeml program from PAML (version 4.9)^84^. We applied the one-ratio model (M0) to estimate overall *d*_*N*_/*d*_*S*_ ratios for each conserved group separately and free ratios neutral model (M1) to estimate *d*_*N*_*/d*_*S*_ ratios for each branch within conserved clades^85^. To study the evolution of PDLP5 and PDLP8, sitewise-analyses of their homologs was carried out. As PDLP5 is specific to Brassicaceae, we added nucleotide sequences for orthologs of AtPDLP5 from NCBI, Phytozome and CoGe databases. Furthermore, additional sequences for orthologs of AtPDLP8 were included in the alignment to improve depth and reliability of the analysis. Multiple sequence alignments of coding nucleotide sequences were constructed with PRANK^82^ and phylogenetic trees were estimated using RAxML^79^ for codeml.

### Syntenic vs tandem duplications

Syntenic and tandem duplications were analyzed using Synmap application in CoGe^86^ using default settings. Tandem duplications were defined as genome regions with at least three to five duplicate genes (Supplementary Data 4). Synteny comparisons were done between *Arabidopsis thaliana* and *Solanum lycopersicum*, *S. lycopersicum* and *Amborella trichopoda*, *A. trichopoda* and *Oryza sativa*, and *Zea mays* and *Oryza sativa*. Tandem duplication results from DAGchainer were collected for each species. The results were filtered based on annotated gene models from selected species. The currently available *Amborella trichopoda* genome is presented only as scaffolds, and the genes were placed to chromosomes based on physical mapping^87^. Scaffolds not assigned to any chromosome were added separately. Thus the location of the *Amborella trichopoda* genes in the genome is only a rough estimate (Fig. 7a).

### Gene conversion analyses

Gene conversion events were estimated from nucleotide sequences for the same 11 species that were analyzed for *d*_*N*_*/d*_*S*_ ratios with GENECONV (version 1.81a)^88^. Analyses were carried out for the main clades of the 11 species. For bCRKs and vCRKs separate analyses were carried out using sequences from the five species used in synteny analyses (*Arabidopsis thaliana*, *Amborella trichopoda, Oryza sativa*, *Solanum lycopersicum*, and *Zea mays*). The largest tandem region of vCRKs in *A. thaliana* chromosome 4 was analyzed separately to validate the results from the analysis with all vCRKs from *A. thaliana*.

### Gene tree reconciliation

Gene tree reconciliation was carried out using DLCpar (version 1.0)^89^ downloaded from https://www.cs.hmc.edu/~yjw/software/dlcpar/. NCBI taxonomy was used as the species tree, downloaded in newick format from PhyloT website, http://phylot.biobyte.de/. Reconciliation was carried out using DLCpar search with 20 prescreening iterations, followed by 1000 search iterations. The solution was visualized in R, using custom scripts and “ape” package.

### Phenomics data analysis

Phenotyping data of T-DNA mutant insertion lines were normalized against the Col-0 data by calculating *Z*-scores, see Bourdais et al.^24^ The standard deviation (SD) over all experiments was calculated for each allele, and in case of several insertion alleles the one with maximum SD was selected. The residuals of the bCRK vs vCRK split in the data were tested for normality using Shapiro’s test. Since the null hypothesis (normality) was rejected with *p* < 0.05 the difference between groups was tested with Wilcox test.

### Transcriptomic analyses

Paired end RNAseq data were collected from the publicly available sequence read archive (SRA) database by fastq-dump.2 (version 2.5.7) for *Arabidopsis thaliana*, *Oryza sativa*, *Solanum lycopersicum*, and *Zea mays*. FastQC (version 0.11.4) (https://www.bioinformatics.babraham.ac.uk/projects/fastqc/) was used to check the quality of the samples. Low-quality reads and bases were removed by Trimmomatic (version 0.36)^90^ with the following options: phred33, TRAILING: 20, and MINLEN: 30. Filtered reads were mapped to gene models from Phytozome version 12, by Kallisto, run in paired end mode (version 0.43.1, --bias and --bootstrap: 200)^91^. Bootstrap samples were averaged (custom R code) and gene expression abundance (transcript per million [TPM]) was estimated by tximport (version 1.2.0)^92^ followed by averaging over biological replicates. Ortholog comparison between species was carried out by grouping the experiments into seven categories, with maximum TPM among experiments representing gene response. Pearson correlation was calculated among orthologs and all other possible pairs.

### Protein expression and purification

An expression construct coding for the *PDLP5* ectodomain (amino acids 1–241) was codon optimized for *Spodoptera frugiperda* and synthesized by Geneart (Thermo Fisher). Using the PfuX7 polymerase^93^, the gene for the *PDLP8*-ECD (1–253) was amplified from *Arabidopsis thaliana* cDNA. The Gibson assembly method^94^ was employed to insert the *PDLP5* and *PDLP8* ectodomain coding sequences into an adapted pFAST-BAC1 vector (Geneva Biotech), providing a C-terminal 2x-STREP-9xHIS tag. *PDLP5* point mutations (C101A, C148A, and C191A) were then introduced as described^95^. Bacmids were generated by transforming the plasmids (confirmed by sequencing) into *Escherichia coli* DH10MultiBac (Geneva Biotech). Virus particles were created by transfecting (Profectin, AB Vector) the bacmids into *Spodoptera frugiperda* SF9 cells (Thermo Fisher). For secreted protein production, *Trichoplusia ni* Tnao38 cells (obtained from Gary W Blissard, Boyce Thompson Institute, Tower Road, Ithaca, NY, USA)^96^ were infected with a viral multiplicity of 1, incubated for 3 days at 22 °C. The protein-containing supernatant was separated from the intact cells by centrifugation and subjected to Ni^2+^-affinity chromatography (HisTrap Excel; GE Healthcare) in buffer A (10 mM Hepes 7.5, 500 mM NaCl). Bound proteins eluted in buffer A supplemented with 500 mM imidazole. The elution fractions were pooled and further purified by StrepII-affinity purification (Strep-Tactin XT Superflow high capacity, IBA) in buffer B (20 mM Tris pH 8.0, 250 mM NaCl, 1 mM EDTA). The column was washed with 5–10 column volumes of buffer B and eluted in buffer B supplemented with 50 mM biotin. The C-terminal 2x-STREP-9xHIS tag was subsequently removed by adding tobacco etch virus (TEV)-protease to the StrepII elution in a 1:100 ratio for 16 h at 4 °C. The 2x-STREP-9xHIS-tag and the HIS-tagged TEV-protease were then separated from the respective ectodomain by an additional Ni^2+^-affinity chromatography step (HisTrap Excel; GE Healthcare). Cleaved PDLP5, PDLP5^C101A^, PDLP5^C148A^, PDLP5^C191A^, and PDLP8 ectodomains were next subjected to preparative size exclusion chromatography using either a HiLoad 26/600 Superdex 200 pg (PDLP5 and PDLP8) or HiLoad 16/600 Superdex 200 pg (PDLP5^C101A^, PDLP5^C148A^, and PDLP5^C191A^) column, equilibrated in 20 mM sodium citrate pH 5.0 and 150 mM NaCl. Monomeric peak fractions were collected and concentrated using an Amicon Ultra (Millipore) filter device. The concentrated monomeric peak fractions of PDLP5, PDLP5^C101A^, PDLP5^C148A^, and PDLP5^C191A^ were additionally subjected to analytical size exclusion chromatography on a Superdex 200 Increase 10/300 GL column (GE Healthcare) equilibrated in 20 mM citrate pH 5.0 and 150 mM NaCl (Supplementary Figure 9, uncropped gel images are available in Supplementary Figure 16).

### Thermostability assay

Twenty microliters reactions consisted of either PDLP5, PDLP5^C101A^, PDLP5^C148A^, and PDLP5^C191A^ ectodomains at a concentration of 1.5 mg/ml in 20 mM citrate pH 5.0, 150 mM NaCl, 10× SYPRO Orange dye (Thermo Fisher), and were mixed in a 384-well ABI PRISM plate (Applied Biosystems). Using a 7900HT Fast Real-Time PCR system SYPRO Orange fluorescence was measured. The reactions were initially incubated for 2 min at 25 °C and then the temperature was increased to 95 °C at a heating rate of 0.5 °C/min. Resulting melting curves were fitted with a Boltzman function using GraphPad Prism and the melting temperatures, *T*_m_, correspond to the first inflection point of the Boltzman fit.

### Isothermal titration calorimetry

ITC experiments were performed at 25 °C using a Nano ITC (TA Instruments, New Castle, USA) with a 1.0 ml standard cell and a 250 μl titration syringe. The PDLP5 ectodomain was gelfiltrated into ITC buffer (20 mM sodium citrate pH 5.0, 150 mM NaCl) and all carbohydrates were resuspended into ITC buffer. The experiments were carried out by injecting 24 times 10 μl of d-+-Mannose (1 mM; Sigma), Pectic Galactan (2 mg/ml; Megazyme), Rhamnogalacturonan (2 mg/ml; Megazyme), polygalacturonic acid (2 mg/ml; Megazyme), Cellohexaose (1 mM; Megazyme), or Arabinohexaose (1 mM; Megazyme) aliquots into PDLP5 (~100 μM) in the cell at 150 s intervals. ITC data for the d-+-mannose experiment were corrected for the heat of dilution by subtracting the mixing enthalpies for titrant solution injections into protein-free ITC buffer. Data were analyzed using the NanoAnalyze program (version 3.5) as provided by the manufacturer.

### Protein crystallization and crystallographic data collection

The PDLP5 ectodomain formed crystals in hanging drops composed of 1 μl of protein solution (70 mg/ml in 20 mM citrate pH 5.0 and 150 mM NaCl) and 1 μl of crystallization buffer (17.5% [w/v] polyethylene glycol 4000, 250 mM (NH_4_)_2_SO_4_) suspended over 800 μl of the latter as reservoir solution. Protein crystals were transferred into crystallization buffer supplemented with 25% (v/v) ethylene glycol, which served as cryoprotectant, and snap frozen in liquid N_2_. PDLP8 crystals (52 mg/ml in 20 mM citrate pH 5.0, 150 mM NaCl) developed in hanging drops containing 17.5 % (w/v) polyethylene glycol 4000, 0.1 M citrate pH 5.5, 20% (v/v) 2-propanol. Crystals were frozen directly in liquid N_2_. For PDLP5 native (*λ* = 1.0 Å) and redundant sulfur SAD (*λ* = 2.079 Å) data were collected to 1.29 Å resolution at beam line PXIII of the Swiss Light Source (SLS), Villigen, Switzerland. A 1.95 Å native dataset of PDLP8 was acquired at the same beam line. Data processing and reduction was done with XDS (version: Jan 2018)^97^.

### Structure solution and refinement

The structure of PDLP5 was solved using the single-anomalous diffraction (SAD) method. Twenty-four S sites corresponding to the 12 disulfide bonds in the PDLP5 crystallographic dimer were located with the program SHELXD^98^, site-refinement and phasing was done in SHARP^99^, and the starting phases were used for automated model building in BUCCANEER^100^ and ARP/wARP^101^. The model was completed in alternating cycles of model correction in COOT^102^ and restrained refinement in Refmac5^103^. The structure of PDLP8 was solved using the molecular replacement methods as implemented in the program PHASER^104^, and using the refined PDLP5 tandem ectodomain as search model. Inspection with MolProbity^105^ revealed excellent stereochemistry for the final models. Structural and surface representations were done in Pymol (http://pymol.soureforge.org) and Chimera^106^.

### Code availability

Scripts for parsing and visualizing data have been deposited in GitHub and can be retrieved from https://github.com/jsalojar/DLCpar_visualize/.

### Reporting Summary

Further information on experimental design is available in the Nature Research Reporting Summary linked to this Article.

## Supplementary Information

Supplementary Information

Description of Additional Supplementary Files

Reporting Summary

Supplementary Data 1

Supplementary Data 2

Supplementary Data 3

Supplementary Data 4

Supplementary Data 5

## Data Availability

Sequence information used in this study is available as Supplementary Data 5. Phylogenetic trees with bootstrap information for 1000 replicates and corresponding sequence alignments have been deposited on Wasabi (http://wasabiapp.org); identifiers are available in the figure legends as web links. Information on used genomic data is available in Supplementary Table 2. Publicly available gene expression data were taken from the Sequence Read Archive (SRA) database; identifiers are listed in Supplementary Data 4. Crystallographic coordinates and structure factors have been deposited with the Protein Data Bank (http://rcsb.org) with accession codes 6GRE (PDLP5) and 6GRF (PDLP8). Refinement statistics are available as Supplementary Table 1. Other materials are available from the corresponding author upon request.
