# Supplementary material for: Mechanistic insights into the evolution of DUF26-containing proteins in land plants
Source: Commun Biol. 2019 Feb 8;2:56. doi: 10.1038/s42003-019-0306-9 (PMC6368629; doi:10.1038/s42003-019-0306-9)
Supplement: Supplementary file 1 — Supplementary Information [file 42003_2019_306_MOESM1_ESM.pdf]

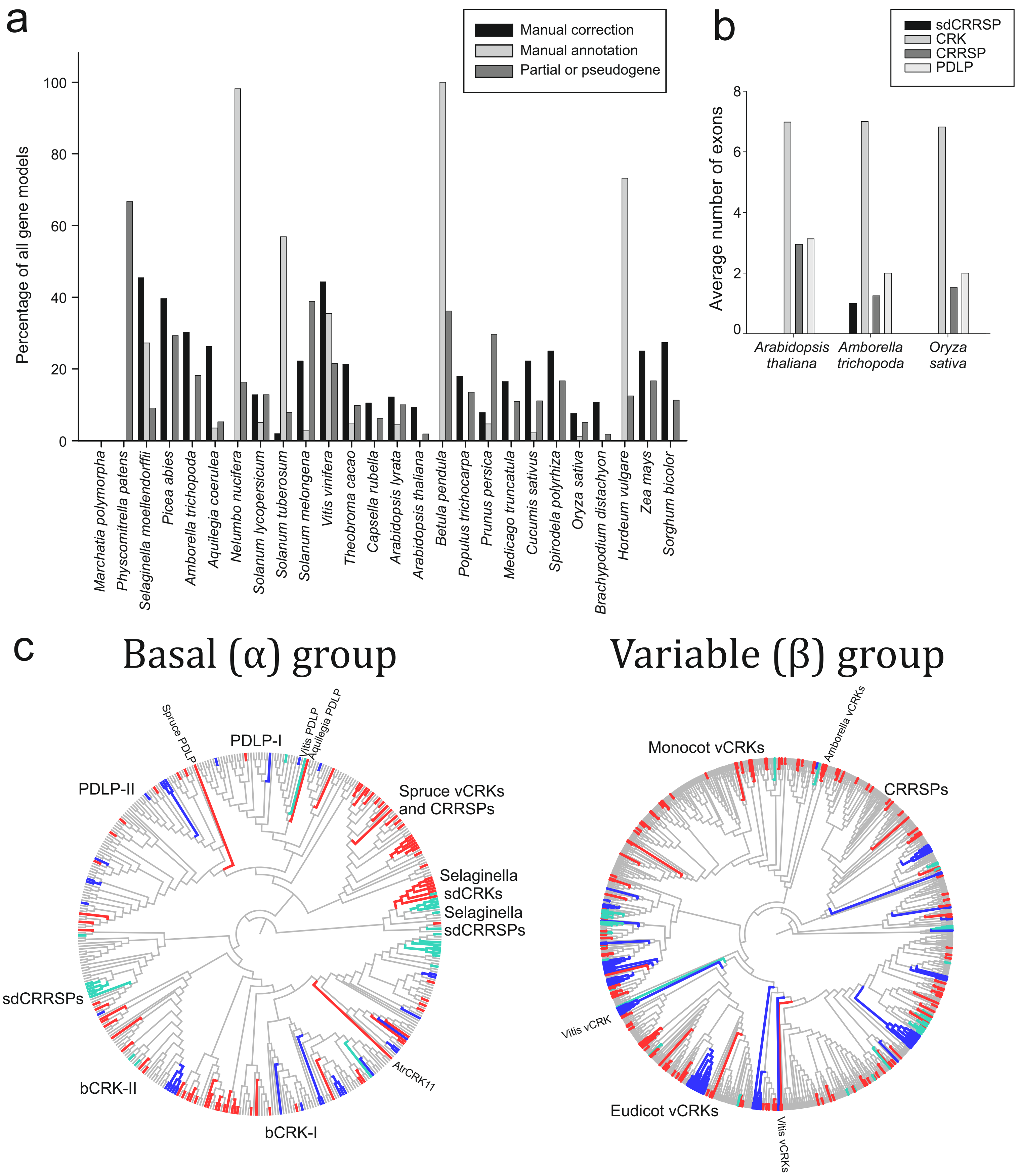

Supplementary Figure 1. Summary of manual gene annotation and correction. a) The number of corrected, manually annotated and partial/pseudo gene models in the studied species. Percentage of corrected gene models is marked with light grey, manually annotated genes with black and genes classified as partial or pseudogenes with dark grey. Silver birch (*Betula pendula*) and sacred lotus (*Nelumbo nucifera*) genes were fully manually annotated, as the gene models were not available when the study was initiated. *Selaginella moellendorffii* and *Vitis vinifera* required highest percentage of manual corrections. The high percentage of pseudogenes in *Physcomitrella patens* is explained by low gene number (two out of three gene models are likely pseudogenes). **b)** Average exon numbers of CRRSPs, PDLPs and CRKs. Average exon numbers were calculated for sdCRRSPs, ddCRRSPs, PDLPs and CRKs in *Amborella trichopoda*, *Arabidopsis thaliana* and *Oryza sativa*. **c)** The amount of curated and manually annotated gene models in basal and variable groups. Corrected (red) and manually annotated (turquoise: species with pre-existing annotations; blue: species without previous annotations) gene models marked in both groups. Corrected or annotated genes can be found in all subgroups within these groups. There are several examples of corrected or previously non-annotated genes that are basal for subgroups, indicating the importance of gene model validation for correct tree topology.

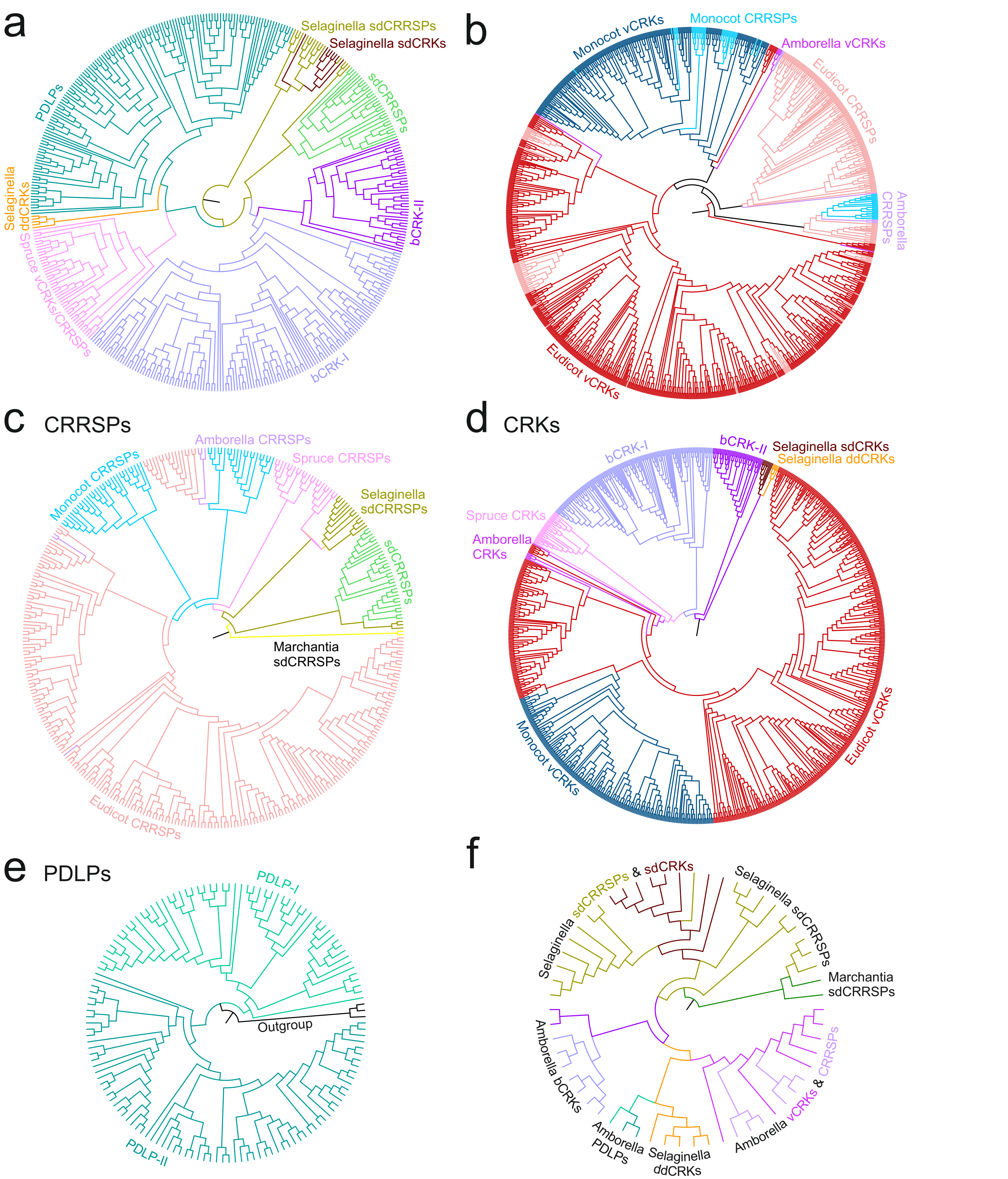

Supplementary Figure 2. Phylogenies of DUF26-containing proteins. a) A phylogenetic maximum-likelihood tree was estimated with full-length sequences for the basal group containing Selaginella sdCRRSPs and CRKs, Norway spruce CRRSPs and CRKs, monocot and eudicot bCRKs and PDLPs. Detailed phylogenetic trees with bootstrap support (1000 replicates) and filtered sequence alignment can be found at http://was.bi?id=wpEHGt. **b)** The phylogenetic maximum-likelihood tree for the variable group contains angiosperm CRRSPs and vCRKs. Tree was estimated using the full-length sequences. Detailed phylogenetic trees with bootstrap support (1000 replicates) and filtered sequence alignment can be found at http://was.bi?id=aIJe_D. Phylogenetic maximum likelihood trees of **c)** CRRSPs **d)** CRKs and **e)** PDLPs. Detailed phylogenetic trees containing gene identifiers as well as bootstrap support (1000 replicates) and filtered sequence alignment can be found at http://was.bi?id=zbIl7i (CRRSPs), http://was.bi?id=i9To8q (CRKs) and http://was.bi?id=Fe1A3A (PDLPs). **f)** Phylogenetic maximum-likelihood tree of all DUF26 genes in *Marchantia polymorpha*, *Selaginella moellendorffii* and *Amborella trichopoda*. Tree is estimated from sequence alignment of full length gene models where the sites with coverage less than 10% have been filtered out. Tree is rooted to sdCRRSPs from *Marchantia polymorpha*. A detailed phylogenetic tree with gene identifiers as well as bootstrap support (1000 replicates) and filtered sequence alignment can be found at http://was.bi?id=VeeQZ6.

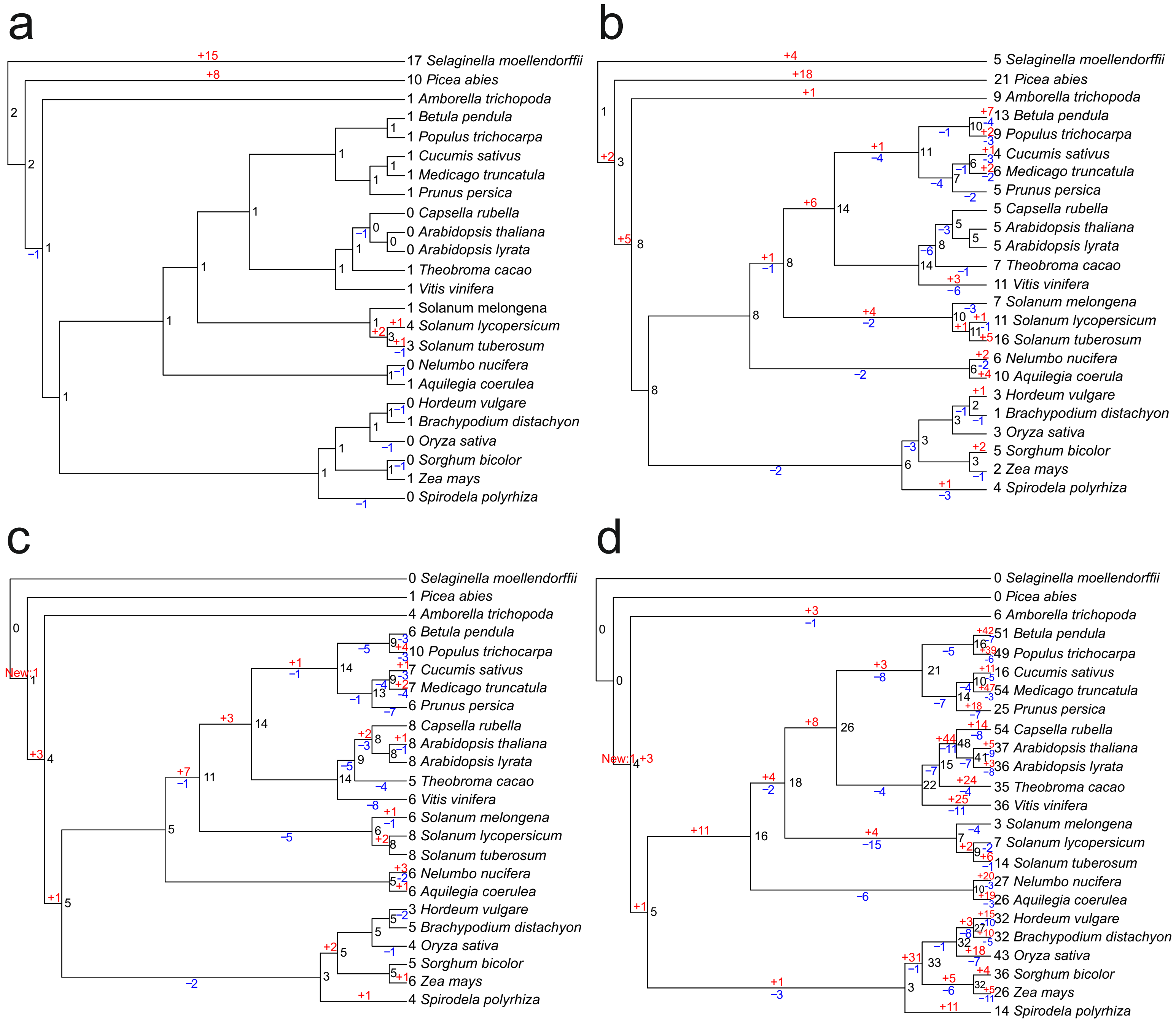

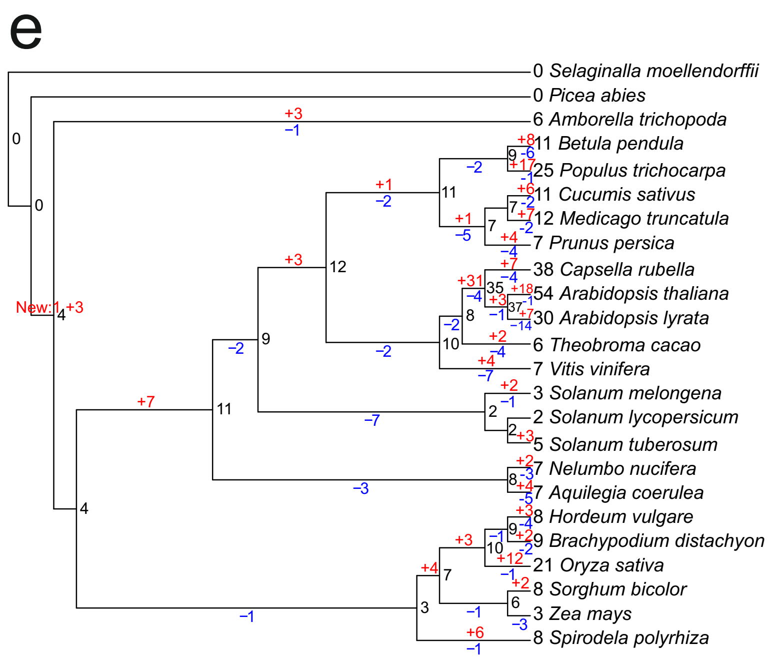

Supplementary Figure 3. Ancestral gene counts for DUF26-containing genes. DLCpar was used for inferring the most parsimonious history of protein groups in the presence of duplications, losses, and incomplete lineage sorting. The panels illustrate ancestral gene counts and lineage-specific expansions in **a)** sdCRRSPs in the basal group, **b)** basal CRKs, **c)** PDLPs, **d)** variable group CRKs and **e)** ddCRRSPs in the variable group. Numbers with black color show the gene counts in the species and their most recent common ancestor. Estimated gene gains are marked with red and losses with blue.

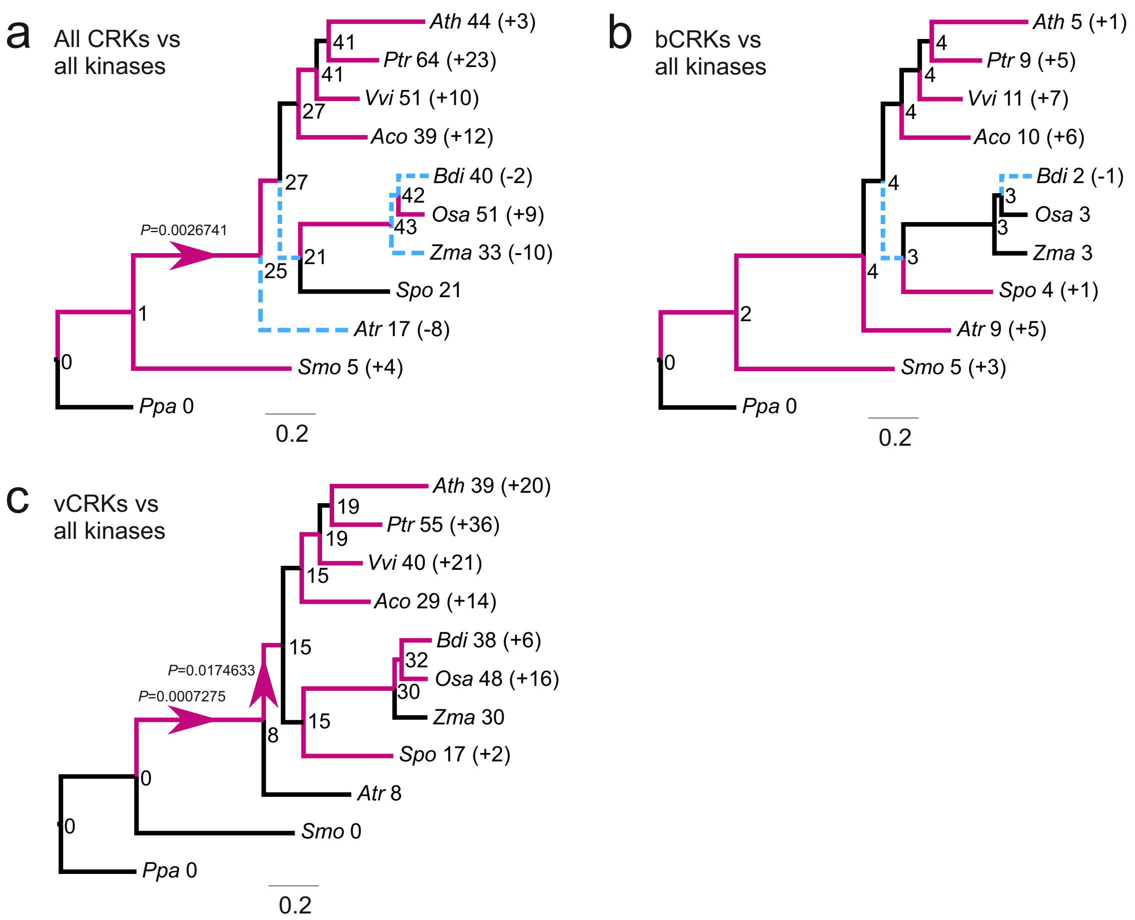

Supplementary Figure 4. Badirate comparisons for evolutionary rates. Analyses were carried out with Badirate for eleven species (*Physcomitrella patens*, *Selaginella moellendorffii*, *Amborella trichopoda*, *Arabidopsis thaliana*, *Populus trichocarpa*, *Vitis vinifera*, *Aquilegia coerulea*, *Spirodela polyrhiza*, *Zea mays*, *Oryza sativa* and *Brachypodium distachyon*). Neutral branches: bold black lines; gene family expansion: bold purple lines; gene family contraction: blue dashed lines. Branches with a significant difference to birth-death model estimated from orthogroup data are marked with arrows. Node labels present the gene family size in ancestral nodes as estimated by Badirate. Tip labels contain species abbreviation and the change in number compared to the most recent ancestral node. **a)** All CRKs compared to all kinases. **b)** bCRKs compared to all kinases. **c)** vCRKs compared to all kinases.

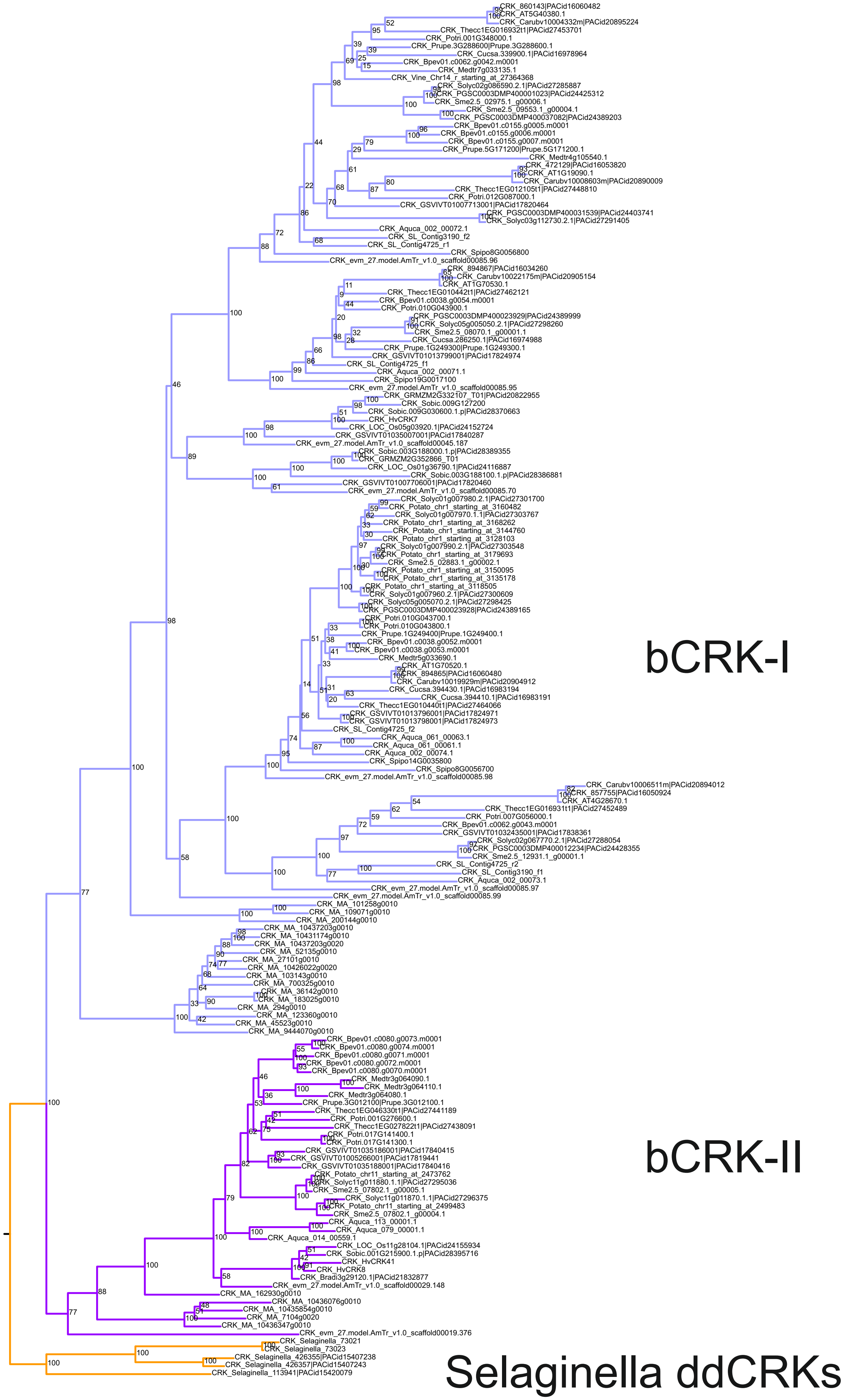

Supplementary Figure 5. Phylogenetic maximum-likelihood tree of bCRKs. The full length sequences belonging to this clade were re-aligned and the alignment was filtered to exclude sites with less than 10% coverage. Bootstrap support is calculated with 1000 replicates. A detailed phylogenetic tree and filtered sequence alignment can be found at http://was.bi?id=6Z7yhQ.

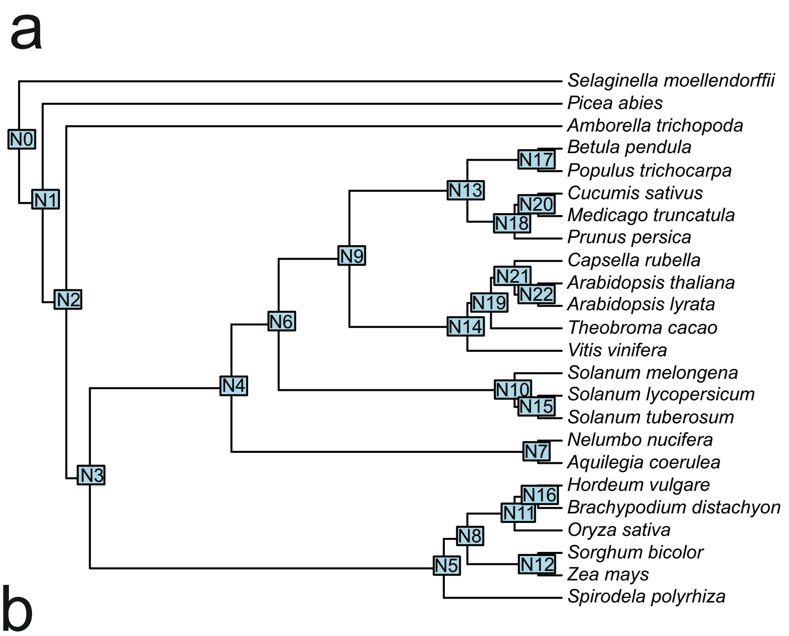

Supplementary Figure 6. Species trees and reconciled phylogenetic trees for DCLpar analyses. a) Species tree for the 24 species where all DUF26-domain genes were comprehensively analyzed. The tree was downloaded from PhyloT. The node labels indicate the speciation event IDs that are used in panels b and c. **b)** Reconciled gene tree for the bCRKs from DCLpar. The node labels provide the timing of the event by referring to the speciation event ID in the species tree. **c)** Reconciled gene tree for the variable group CRRSPs from DLCpar. The node labels provide the timing of the event by referring to the speciation event ID in the species tree.

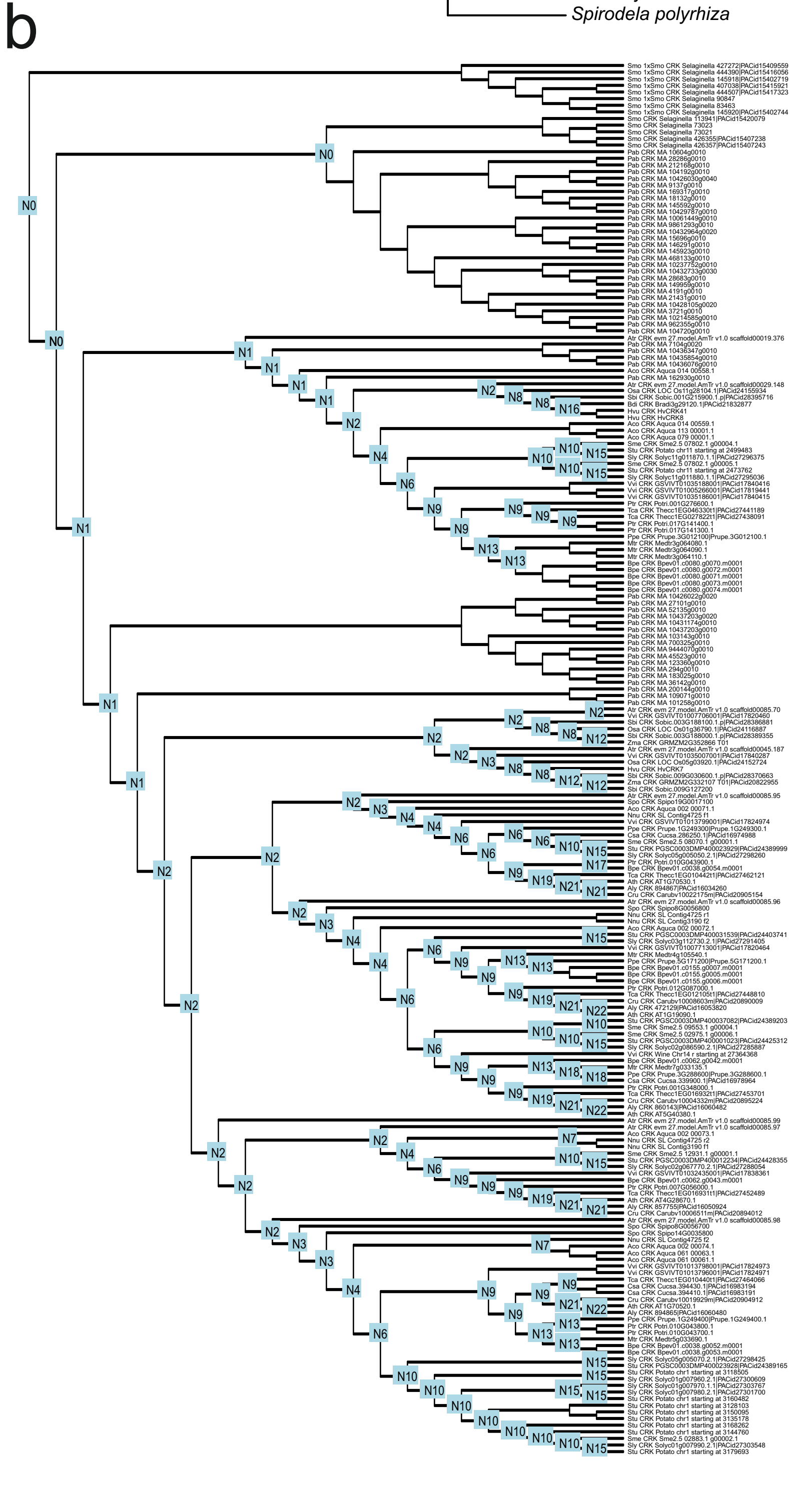

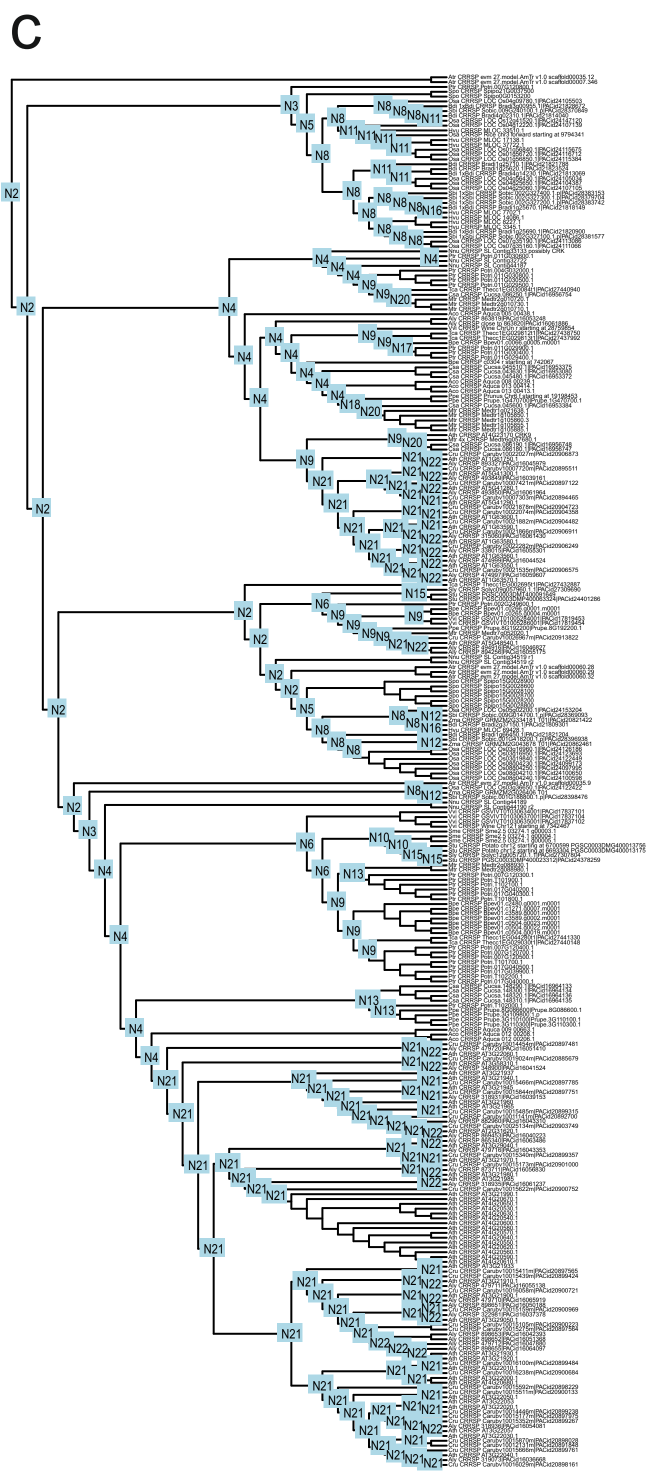

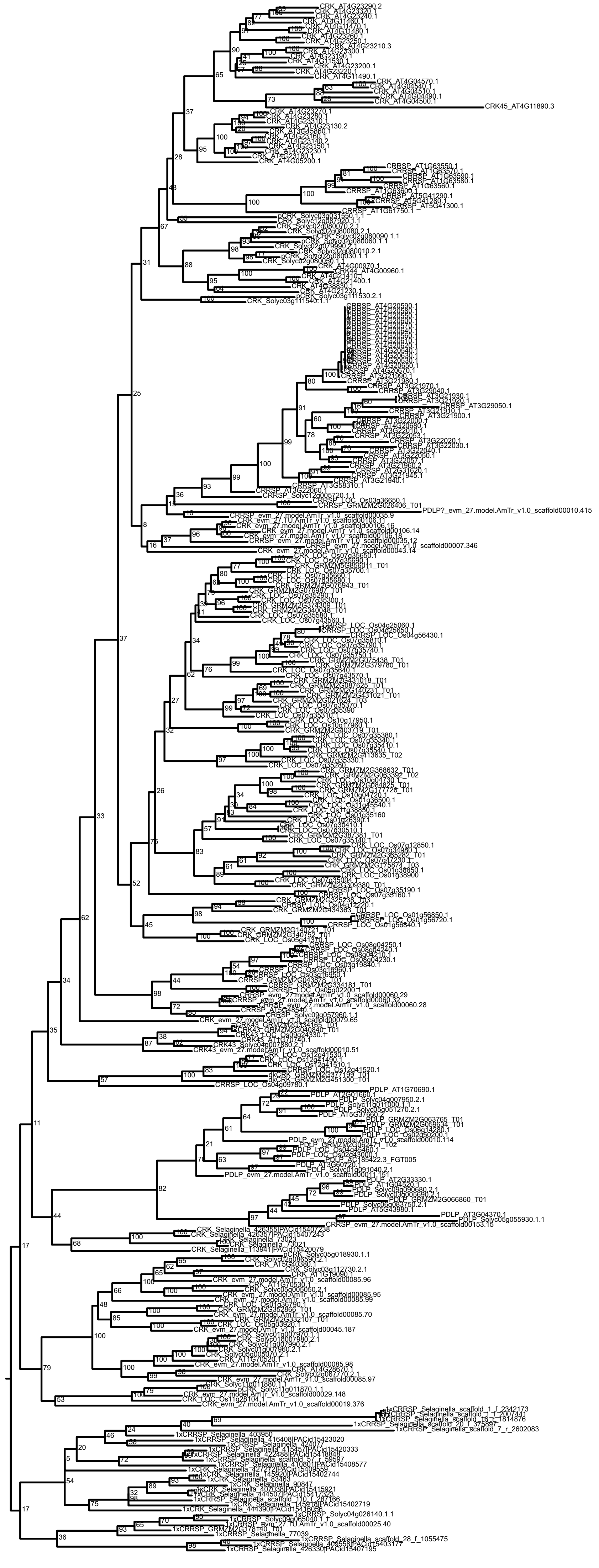

Supplementary Figure 7. Phylogenetic maximum-likelihood tree of 5 species used in segmental duplication analyses and *Selaginella moellendorffii* as outgroup. The tree includes DUF26 genes from *Amborella trichopoda*, *Solanum lycopersicum*, *Arabidopsis thaliana*, *Oryza sativa*, *Zea mays* and *Selaginella moellendorffii*. The full length gene models were used for the sequence alignment and the sites with less than 10% coverage were filtered out. Bootstrap support is calculated with 1000 replicates. A detailed phylogenetic tree and filtered sequence alignment can be found at http://was.bi?id=2NeJCb.

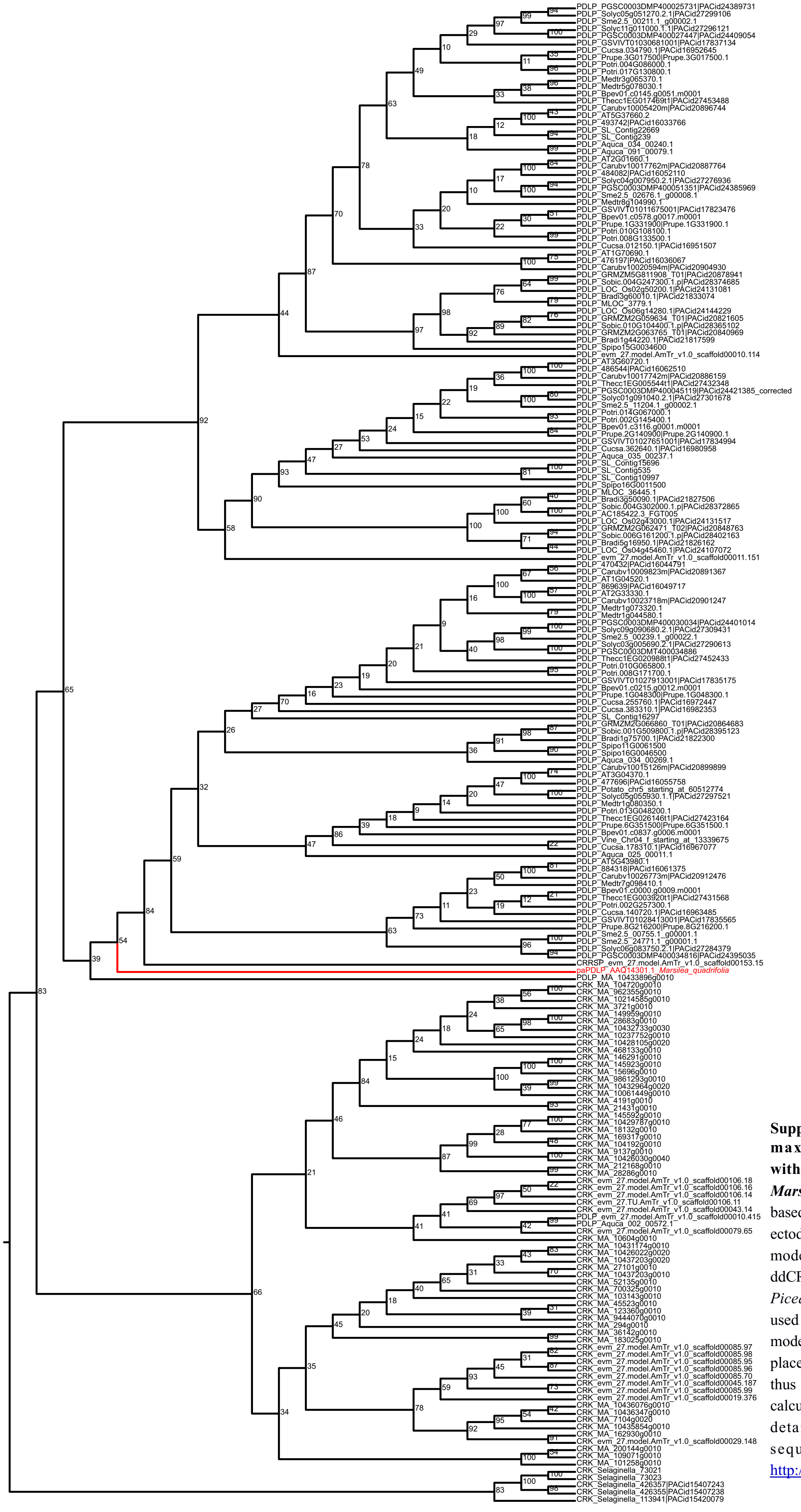

Supplementary Figure 8. Phylogenetic maximumlikelihood tree of PDLPs with possible partial PDLP from *Marsilea quadrifolia*. The phylogenetic tree is based on the sequence covering the part of ectodomain that is present in the partial gene model from *Marsilea quandrifolia*. The ddCRKs from *Selaginella moellendorffii*, *Picea abies* and *Amborella trichopoda* were used as outgroup for PDLPs. The partial gene model from fern *Marsilea quadrifolia* is placed close to the root of PDLP clade and thus could be a PDLP. Bootstrap support is calculated with 1000 replicates. A detailed phylogenetic tree and filtered sequence alignment can be found at http://was.bi?id=usJEbx.

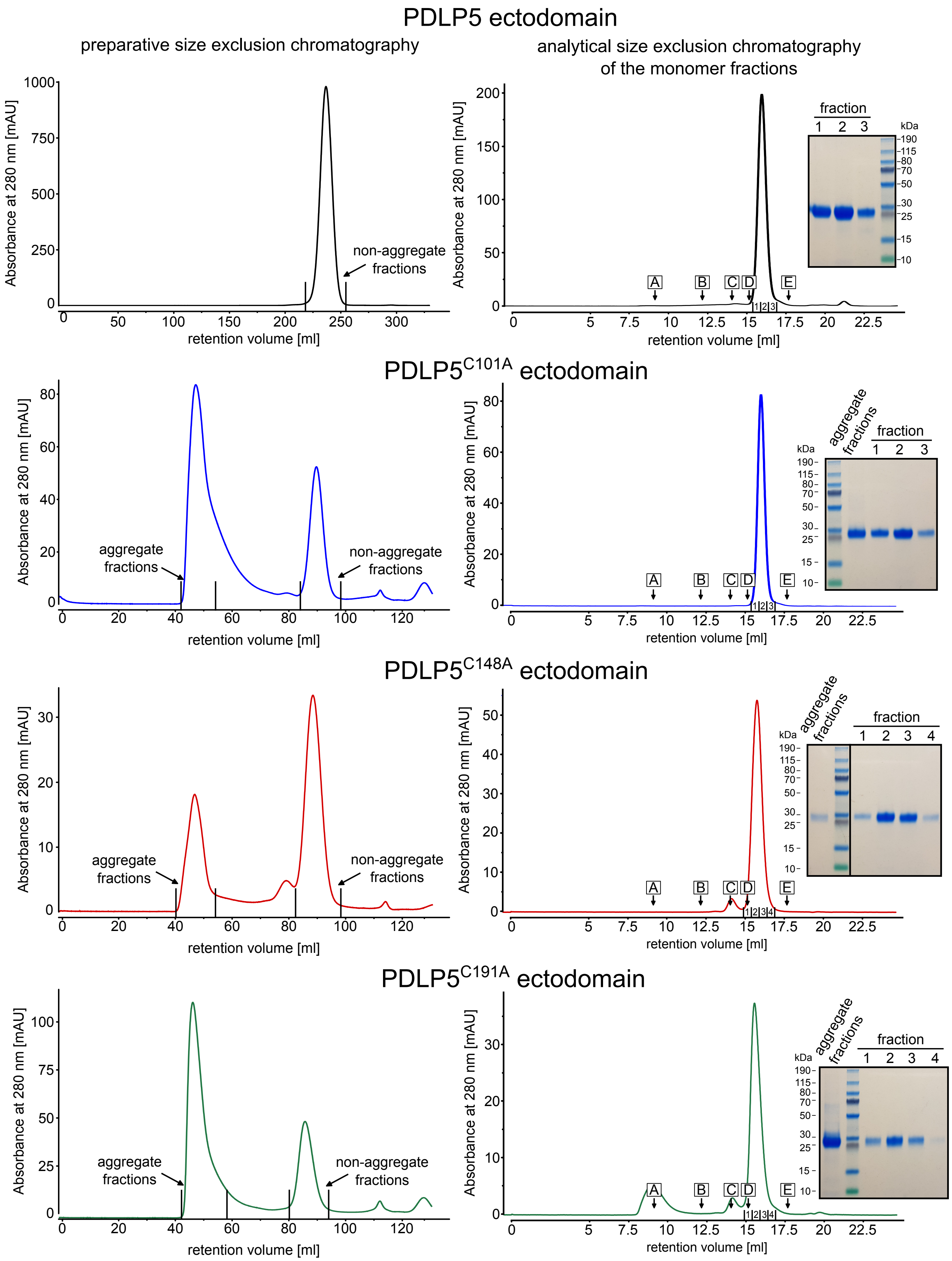

Supplementary Figure 9: Mutation of disulfide bridge forming cysteines in PDLP5 results in protein aggregation. PDLP5, PDLP5^{C101A}, PDLP5^{C148A} and PDLP5^{C191A} ectodomains were subjected to preparative size exclusion chromatography (left). Non-aggregated fractions were combined and subjected to analytical size exclusion chromatography (right). Molecular mass standards: A = Thyroglobulin, 669 kDa B = Aldolase, 158 kDa; C = Conalbumin, 75 kDa; D = Ovalbumin, 44 kDa; E = Ribonuclease A, 13.7 kDa. Non-cropped gel images are shown in Supplementary Figure 16.

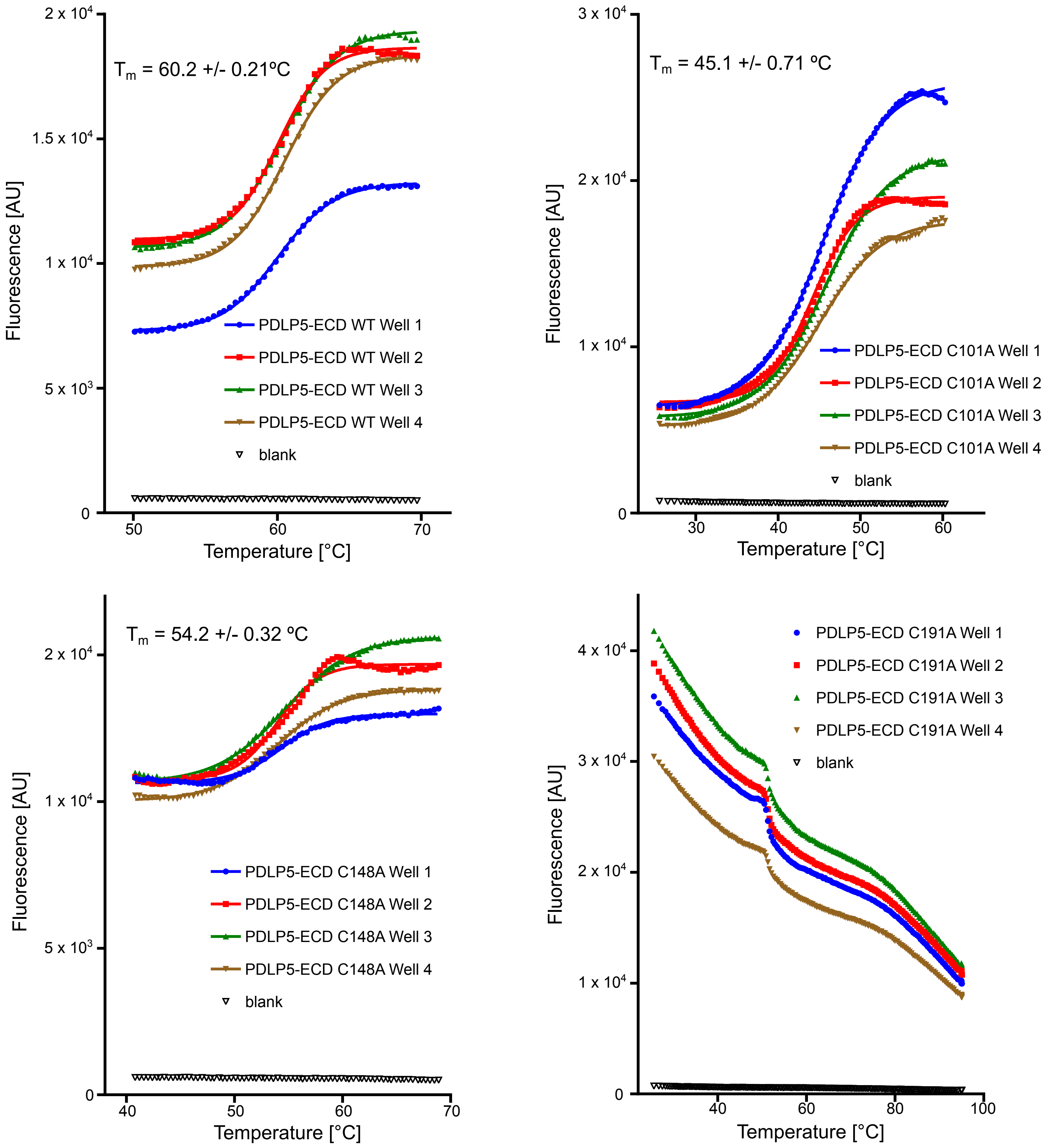

Supplementary Figure 10: Mutations in disulfide bridge forming residues in PDLP5 result in lower protein stability: Melting curves (4 replicates in green, brown, red and blue) of PDLP5, PDLP5^{C101A}, PDLP5^{C148A}, PDLP5^{C191A} ectodomains and of the blank without protein (blank measurements for PDLP5, PDLP5^{C101A}, PDLP5^{C148A} are the same as the experiments were carried out together). For PDLP5, PDLP5^{C101A}, PDLP5^{C148A} ectodomains average melting temperatures are given +/- SDM (n=4). PDLP5^{C191A} was unstable at the given conditions and no melting curve could be acquired.

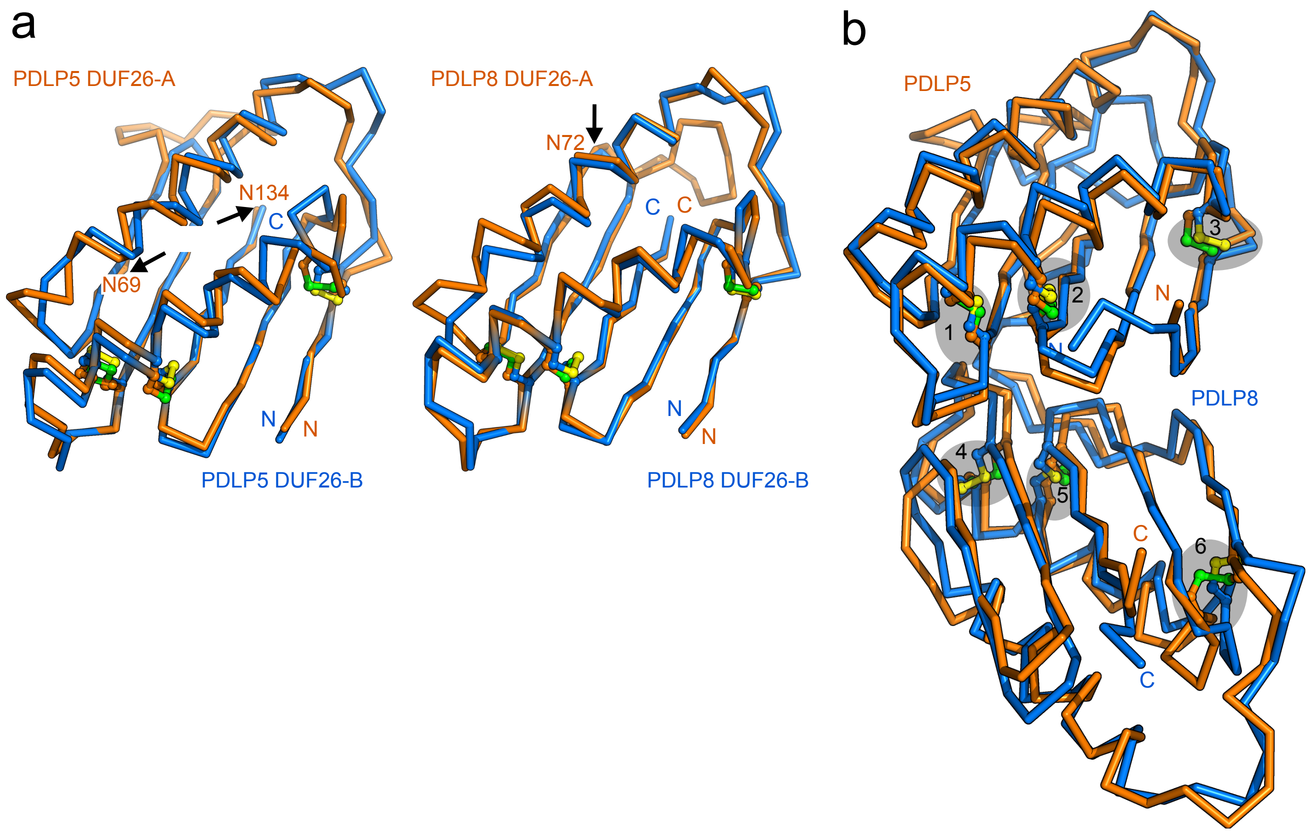

Supplementary Figure 11: Structural comparisons of PDLP5 and PDLP8 DUF26 domains reveal a high de gree of structural similarity (a) Superimposition of the DUF26-A (orange; Cα trace) and the DUF26-B (blue; Cα trace) domains of PDLP5 (left; r.m.s.d. is ~1.6 Å comparing 89 corresponding Cα atoms) and PDLP8 (right; r.m.s.d. is ~1.2 Å comparing 89 corresponding Cα atoms) demonstrate the structural similarity of DUF26-A and DUF26-B domains. Glycosylated asparagines are indicated by an arrow **(b)** Structural superposition of PDLP5 (orange, shown as Cα trace) and PDLP8 (blue) reveals a high degree of overall structural similarity (r.m.s.d. is ~1.6 Å comparing 198 corresponding C α atoms), and a conserved pattern of disulfide bridges (grey highlights). The disulfide bridges in PDLP8 are: 1 (Cys89-Cys98), 2 (Cys101-Cys126), 3 (Cys34-Cys113), 4 (Cys191- Cys200), 5 (Cys203-Cys228) and 6 (Cys148-Cys215). Disulfide bridges are depicted in bonds representation (PDLP5 in yellow, PDLP8 in green).

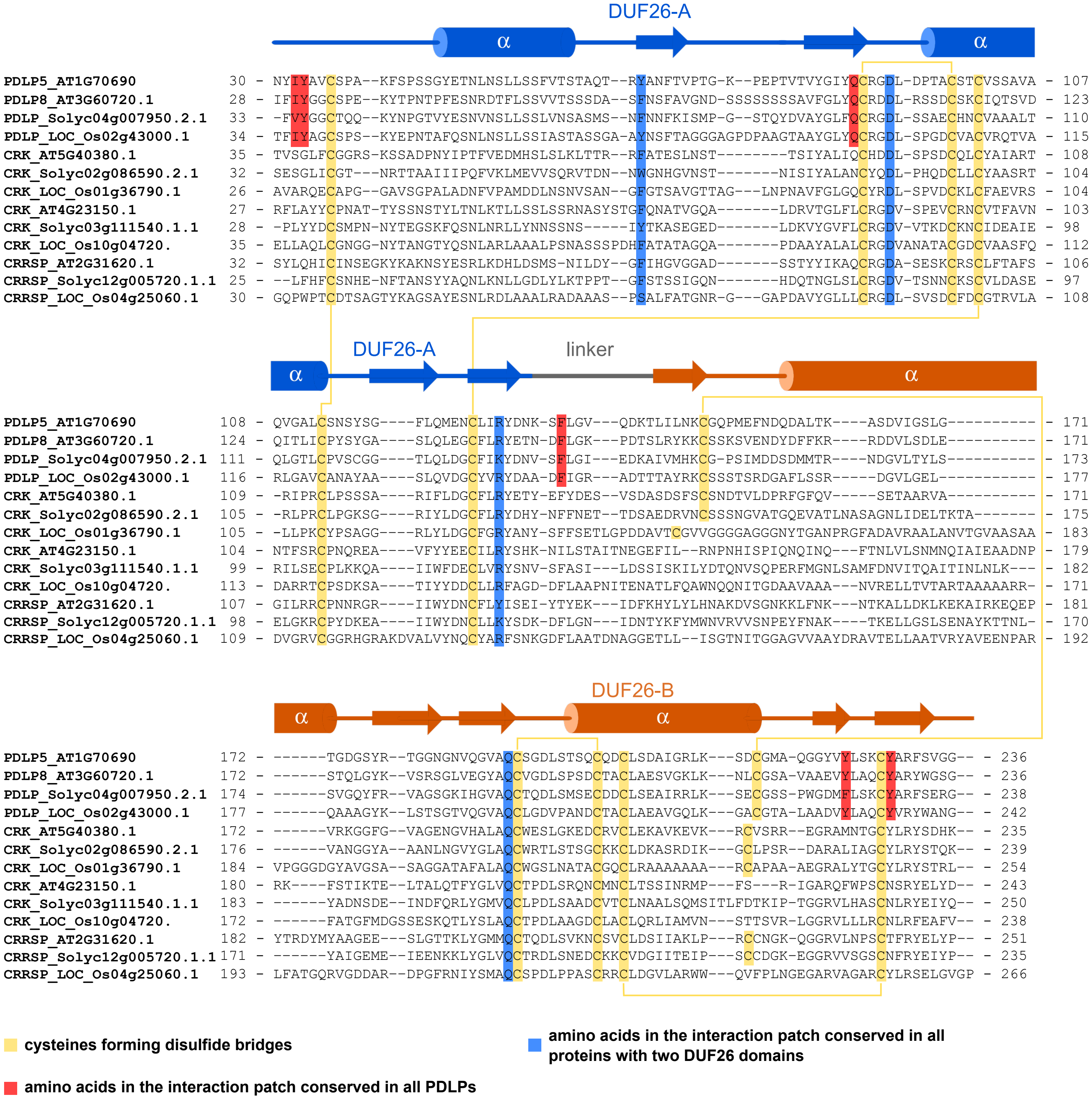

Supplementary Figure 12: Cysteines forming disulfide bonds and residues involved in the interaction of DUF26-A and DUF26-B domains are conserved in bCRKs, vCRKs, CRSPs and PDLPs. A set of PDLPs, bCRKs, vCRKs and CRRSPs were selected based on the structure and their sequences were aligned with MUSCLE[1]. The result shows the conservation of amino acids present in the interaction patch of DUF26-A and DUF26-B in either PDLP5s (red highlight) or all double DUF26 containing proteins (highlight in blue). Cysteines and disulfide bridges are highlighted in yellow. A secondary structure assignment of the DUF26-A (blue) and DUF26-B domains[2] is given above the sequences.

a

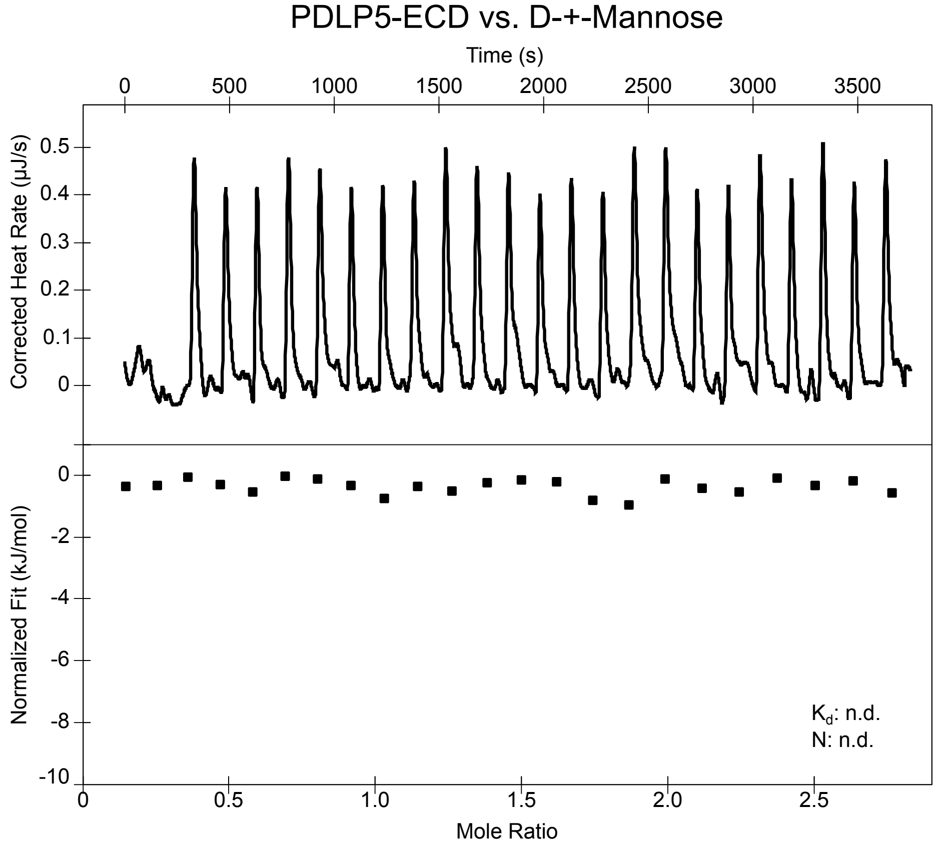

b

cell wall occuring sugars tested for binding to PDLP5-ECD by ITC

Pectic Galactan	no binding detected
Rhamnogalacturonan	no binding detected
Polygalacturonic Acid	no binding detected
Cellohexaose	no binding detected
Arabinohexaose	no binding detected

c

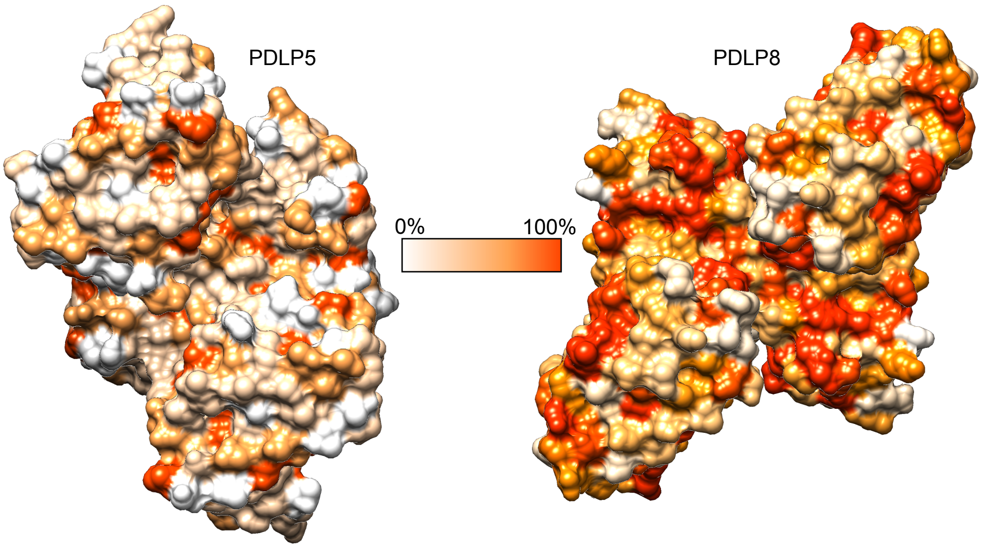

Supplementary Figure 13: The PDLP5 ectodomain does not bind mannose or other cell wall derived sugars and PDLP5 as well as PDLP8 surface exposed residues are not widely conserved. (a) Mannose was titrated into a cell containing the PDLP5 ectodomain in an isothermal titration calorimetry (ITC) assay (n.d., no binding detected). **(b)** ITC experiments were carried out to test binding of plant cell wall sugars to the isolated PDLP5 ectodomain. **(c)** The conservation of amino acids is depicted on the surface of PDLP5 or PDLP8, respectively.

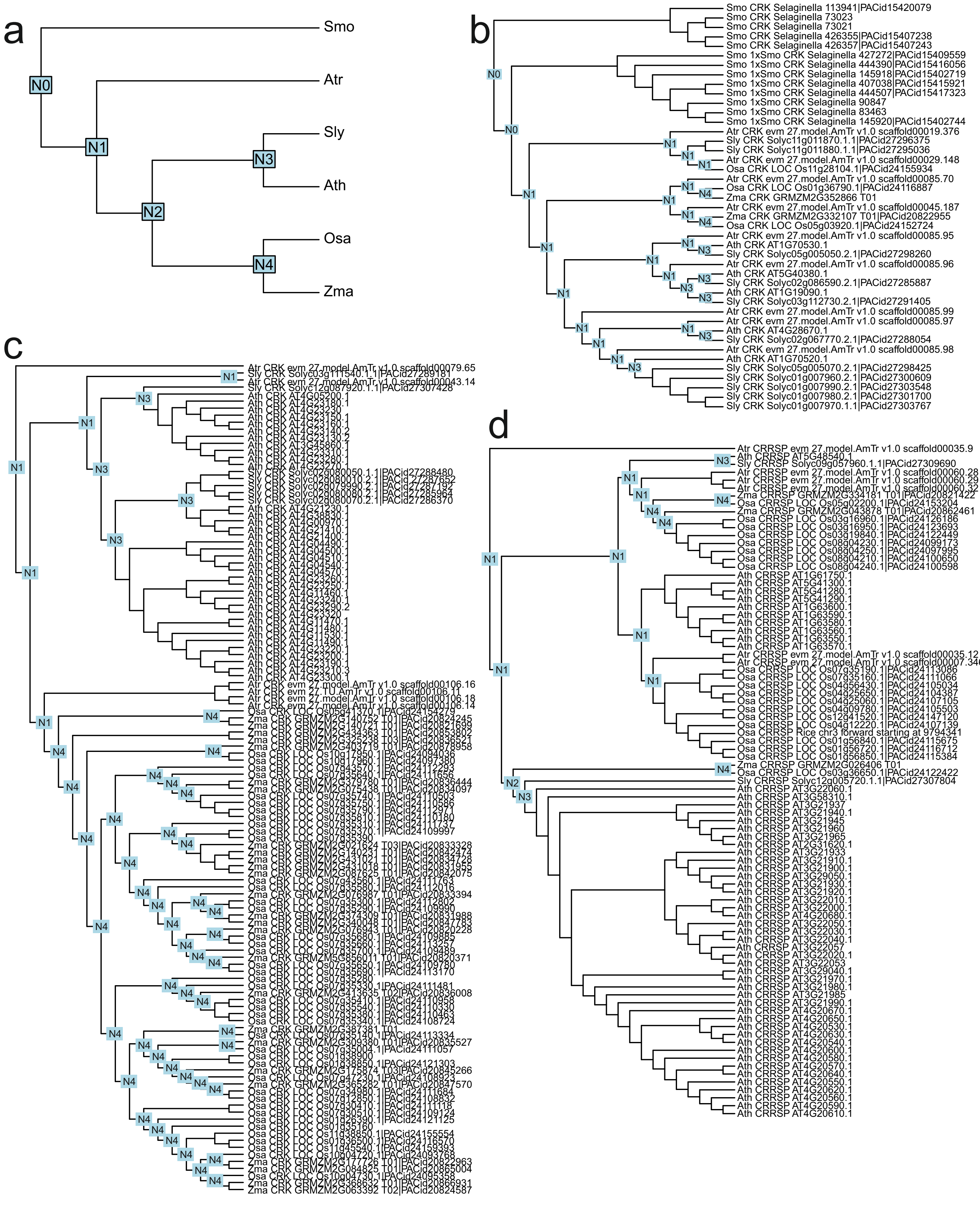

Supplementary Figure 14. Gene tree reconciliation of the five species used in segmental duplication analyses. a) Species tree of the five species in the analyses. The phylogeny was downloaded from PhyloT, and the node labels indicate the speciation event ID. These IDs are used in panels b-d. The reconciled gene trees were estimated with DLCpar for **b)** bCRKs, **c)** vCRKs and **d)** ddCRRSPs. The node labels provide the timing of the split by referring to the speciation event ID in the species tree (panel a). *Selaginella moellendorffii* was used as outgroup.

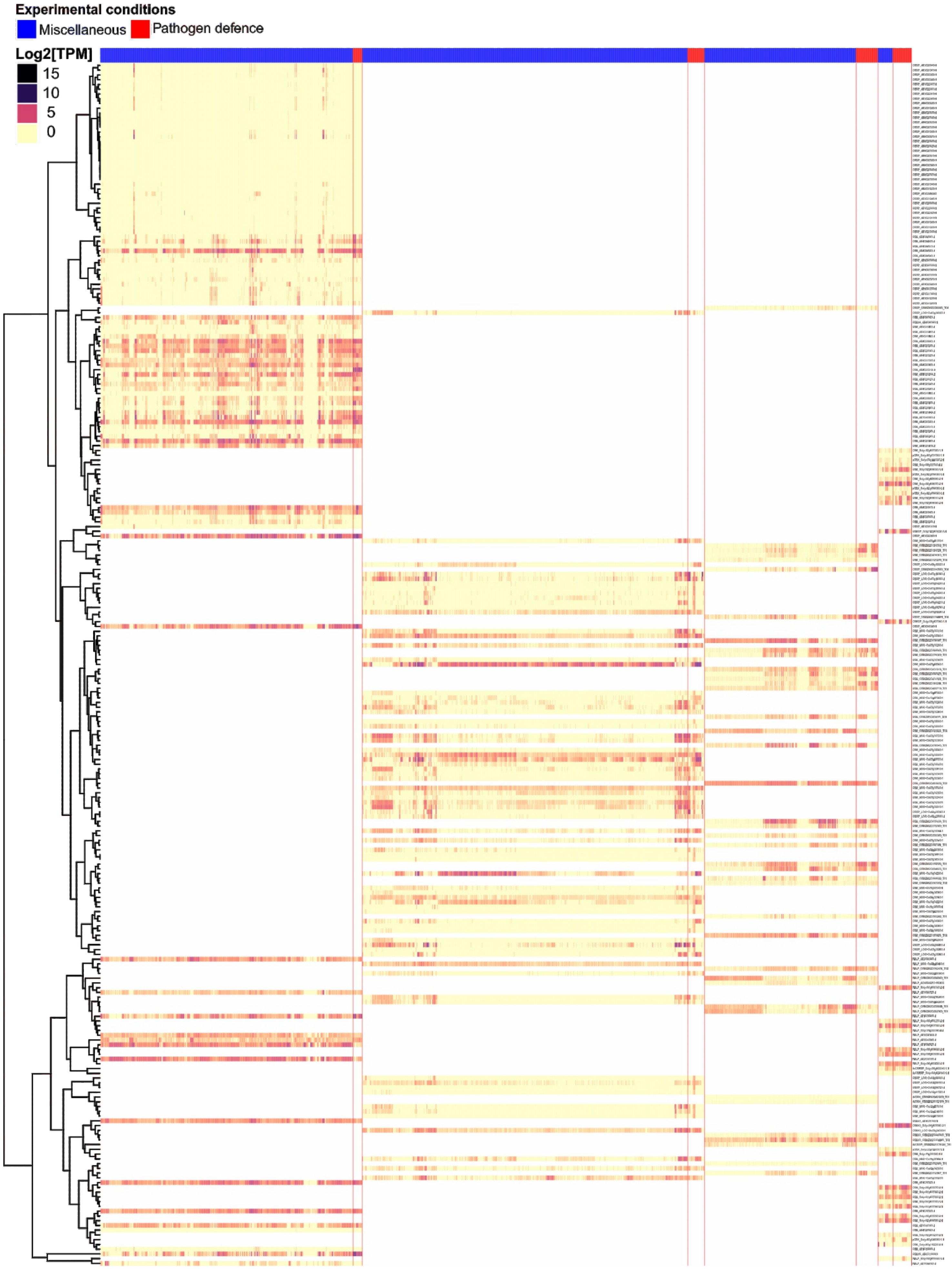

Supplementary Figure 15. The DUF26 genes show transcriptional response to several stress treatments. Heatmap illustrating transcriptional response of DUF26 genes from *Arabidopsis thaliana*, *Oryza sativa*, *Zea mays* and *Solanum lycopersicum*. The dendrogram shows a phylogenetic tree of the 253 DUF26-containing genes (rows) in the four species. The columns represent the RNAseq experiments from Sequence Read Archive (see Supplementary Data 4; accession numbers not shown here for clarity), categorized into pathogen defence (red highlight) and miscellaneous (blue). The heatmap colors represent the $\log_2$(TPM) values, as illustrated by the color key. The NA values are displayed with white color.

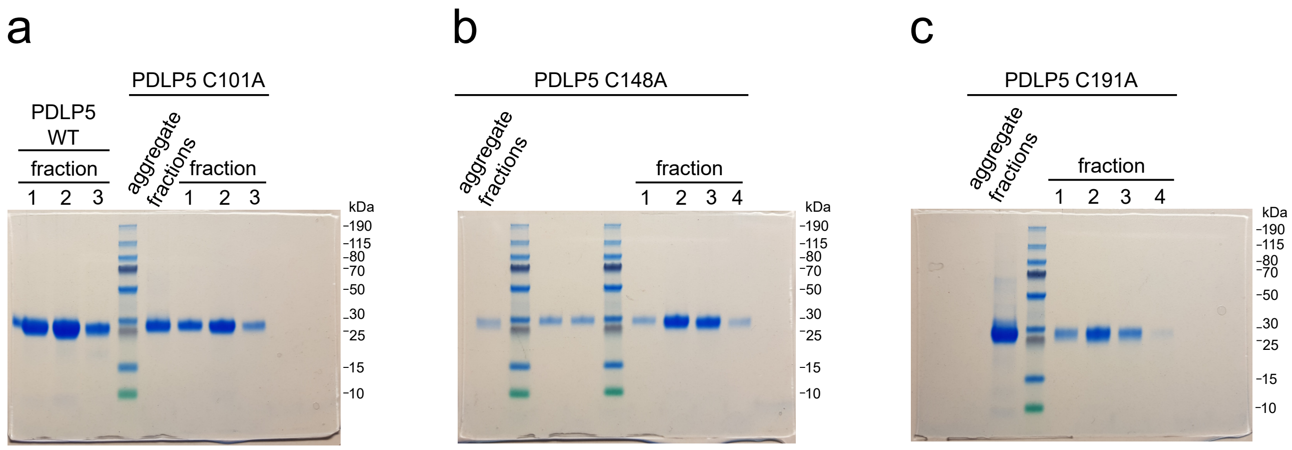

Supplementary Figure 16: Non-cropped gel images of gels shown in Supplementary Figure 9. Whole gel images of the **a)** PDLP5 WT and PDLP5 C101A, **b)** PDLP5 C148A and **c)** PDLP5 C191A ectodomain proteins.

Supplementary Table 1. **Data collection, phasing and refinement statistics**

	**PDLP5** *sulfur SAD**	**PDLP5** *native**	**PDLP8** *native**
**Data collection**			
Space group	*P1*	*P1*	$P3_2\,2\,1$
Cell dimensions			
a, *b*, *c* (Å)	41.81, 48.05, 62.24	41.76, 47.97, 62.19	143.86, 143.86, 59.72
α, β, γ (°)	97.68, 102.74, 99.90	97.70, 102.72, 99.86	90, 90, 120
Resolution (Å)	48.91 – 2.3 (2.36 – 2.30)	37.77 – 1.29 (1.32 – 1.29)	19.91 – 1.95 (2.02 – 1.95)
R_{meas}#	0.004 (0.10)	0.049 (1.29)	0.319 (3.62)
I/σI#	77.5 (33.5)	12.5 (1.1)	8.82 (1.0)
CC (1/2)#	99.9 (99.9)	99.9 (55.7)	99.8 (64.7)
Completeness (%)#	91.2 (83.6)	94.5 (89.4)	100.0 (99.9)
Redundancy#	28.2 (28.5)	3.6 (3.7)	20.1 (20.1)
**Refinement**			
Resolution (Å)		37.77 – 1.29	19.91 – 1.95
No. reflections		103,379	49,227
$R_{work/}\ R_{free}$$		0.18/0.21	0.23/0.26
No. atoms			
protein		3,201	4,821
ligands		153	28
solvent		310	193
Res. B-factors$			
protein		23.1	42.7
ligands		47.5	70.1
solvent		32.3	43.6
R.m.s deviations$			
Bond lengths (Å)		0.012	0.015
Bond angles (°)		1.58	1.36
Ramachandran favored (%)		98.5	97.24
Ramachandran allowed (%)		1.23	2.76
Ramachandran outliers (%)		0.25	0
PDB - ID		**6GRE**	**6GRF**

#as defined XDS[3] or $in Refmac5[4], respectively. *Data were collected from one crystal per experiment.

Supplementary Table 2. Genome version information and references for plant genomes used in phylogenetic analyses.

Species	Genome annotation version
Amborella trichopoda[5]	v1.0
Aquilegia coerulea[6]	v1.1
Arabidopsis lyrata[7]	v1.0
Arabidopsis thaliana[8,9]	TAIR10
Betula pendula[10]	v1.0
Brachypodium distachyon[11]	v3.0
Capsella rubella[12]	v1.0
Chlamydomonas reinhardtii[13]	v5.5
Coccomyxa subellipsoidea[14]	v2.0
*Cucumis sativus**	v1.0
Hordeum vulgare[15]	v2.2
Klebsormidium flaccidum[16]	v1
Marchantia polymorpha[17]	v3.1
Medicago truncatula[18,19]	Mt4.0v1
Micromonas pusilla[20]	v3.0
Nelumbo nucifera[21]	v1
Oryza sativa[22,23]	v7
Ostreococcus lucimarinus[24]	v2.0
Physcomitrella patens[25]	v3.0
Picea abies[26]	v1.0
Populus trichocarpa[27]	v3.0
Prunus persica[28]	v1.0
Selaginella moellendorffii[29]	v1.0
Solanum lycopersicum[30]	iTAG2.4
Solanum melongena[31]	r2.5.1
Solanum tuberosum[32]	v3.4
Sorghum bicolor[33]	v2.1
Spirodela polyrhiza[34]	v1
Theobroma cacao[35]	v1.1
Vitis vinifera[36]	Genoscope.12X
Volvox carteri[37]	v2.0
Zea mays[38]	AGPv3

*No reference publication available, data retrieved from Phytozome[39].

Supplementary Table 3. Description and identifiers for phylogenetic maximum-likelihood trees deposited in Wasabi (http://wasabiapp.org).

Description	Figure reference	Wasabi ID	Wasabi URL
Phylogenetic tree for DUF26-containing proteins	Figure 2	IaroPa	http://was.bi?id=IaroPa
Phylogenetic tree for the basal group of DUF26-containing proteins	Figure 2, Supplementary Figure 2a	wpEHGt	http://was.bi?id= wpEHGt
Phylogenetic tree for the variable group of DUF26-containing proteins	Figure 2, Supplementary Figure 2b	alJe_D	http://was.bi?id=alJe_D
Phylogenetic tree for CRRSPs	Supplementary Figure 2c	zbIl7i	http://was.bi?id=zbIl7i
Phylogenetic tree for CRKs	Supplementary Figure 2d	i9To8q	http://was.bi?id=i9To8q
Phylogenetic tree for PDLPs	Supplementary Figure 2e	Fe1A3A	http://was.bi?id=Fe1A3A
Phylogenetic tree of all DUF26 genes in *Marchantia polymorpha*, *Selaginella moellendorffii* and *Amborella trichopoda*.	Supplementary Figure 2f	VeeQZ6	http://was.bi?id=VeeQZ6
Phylogenetic tree of bCRKs.	Supplementary Figure 5	6Z7yhQ	http://was.bi?id=6Z7yhQ
Phylogenetic tree for five species used in segmental duplication analyses with *Selaginella moellendorffii* as outgroup.	Supplementary Figure 7	2NeJCb	http://was.bi?id=2NeJCb
Phylogenetic tree of PDLPs with possible partial PDLP from *Marsilea quadrifolia*.	Supplementary Figure 8	usJEbx	http://was.bi?id=usJEbx

Supplementary Note 1

Identification and annotation of DUF26 genes

We selected 32 plant species representing major plant lineages for which high-quality genome assemblies are available and retrieved 1656 DUF26-containing gene models (Figure 1a, Supplementary Table 1). 322 gene models required correction, demonstrating the necessity of manual validation of gene family datasets (Supplementary Figure 1). Furthermore, we searched and identified 268 gene models *de novo* from genomic sequences (see Methods). Partial gene models and pseudogenes were excluded resulting in 1409 high-quality models included in subsequent analyses. To confirm that DUF26 is specific to land plants we explored the genomes of the diatom *Phaeodactylum tricornutum*, five algae species, the charophyte *Klebsormidium flaccidum*, as well as fungi, insects and vertebrates (Figure 1a).

Supplementary Note 2

Construction of the phylogenetic trees

Phylogenetic trees were constructed using full length amino acid sequences translated from gene models with intact DUF26 domains. As a result of the different domain compositions only the DUF26-containing region aligned across all sequences. Due to high sequence divergence CRCKs, DUF26-containing gene models from bryophytes and monocot CRKs with a different kinase domain were excluded from the phylogenetic analyses. Amino acid sequence alignments were filtered to reduce the number of very low coverage positions in the alignments.

Supplementary Note 3

Conserved sites specific to α- and β-groups

While most conserved positions within DUF26-A and -B are either conserved in all DUF26-containing proteins or specific to individual types, we identified conserved sites specific to α- or β-groups (Figure 2c).Some conserved positions surrounding the main cysteine motif distinguish members of the α-group from the β-group. These features for α-group include a leucine or isoleucine residue in the fourth position after the first cysteine in the DUF26-A and the position of the fourth cysteine in the DUF26-B (Figure 2c). Similarly**,** a conserved threonine following the first cysteine in DUF26-B (Figure 2c) is specific to β-group.

Supplementary References:

1. Edgar, R.C. MUSCLE: multiple sequence alignment with high accuracy and high throughput. *Nucleic Acids Res.* **32**, 1792-1797 (2004).
2. Kabsch, W. & Sander, C. Dictionary of protein secondary structure: pattern recognition of hydrogen-bonded and geometrical features. *Biopolymers* **22**, 2577-2637 (1983).
3. Kabsch, W. Automatic processing of rotation diffraction data from crystals of initially unknown symmetry and cell constants. *J. Appl. Crystallogr.* 26, 795-800 (1993).
4. Murshudov, G. N., Vagin, A. A. & Dodson, E. J. Refinement of macromolecular structures by the maximum-likelihood method. *Acta Crystallogr. D Biol. Crystallogr.* **53**, 240-255 (1997).
5. Amborella Genome Project. The Amborella genome and the evolution of flowering plants. *Science* **342**, 1241089 (2013).
6. Filiault, D.L. *et al*. The *Aquilegia* genome provides insight into adaptive radiation and reveals an extraordinarily polymorphic chromosome with a unique history. *eLife* 7:e36426 (2018).
7. Hu, T.T. *et al*. The *Arabidopsis lyrata* genome sequence and the basis of rapid genome size change. *Nat. Genet*. **43**, 476-481 (2011).
8. Arabidopsis Genome Initiative. Analysis of the genome sequence of the flowering plant *Arabidopsis thaliana*. *Nature* **408**, 796-815 (2000).
9. Lamesch, P. *et al*. The Arabidopsis Information Resource (TAIR): improved gene annotation and new tools. *Nucleic Acids Res*. **40**, D1202-1210 (2012).
10. Salojärvi, J. *et al*. Genome sequencing and population genomic analyses provide insights into the adaptive landscape of silver birch. *Nat. Genet*. **49**, 904-912 (2017).
11. International Brachypodium Initiative. Genome sequencing and analysis of the model grass *Brachypodium distachyon*. *Nature* **463**, 763-768 (2010).
12. Slotte, T. *et al*. The *Capsella rubella* genome and the genomic consequences of rapid mating system evolution. *Nat. Genet*. **45**, 831-835 (2013).
13. Merchant, S.S. *et al*. The Chlamydomonas genome reveals the evolution of key animal and plant functions. *Science* **318**, 245-250 (2007).
14. Blanc, G. *et al*: The genome of the polar eukaryotic microalga *Coccomyxa subellipsoidea* reveals traits of cold adaptation. *Genome Biol*. **13**, R39 (2012).
15. Mascher. M. *et al*. A chromosome conformation capture ordered sequence of the barley genome. *Nature* **544**, 427-433 (2017).
16. Hori, K. *et al*. *Klebsormidium flaccidum* genome reveals primary factors for plant terrestrial adaptation. *Nat. Commun*. **5**, 3978 (2014).
17. Bowman, J.L. *et al***.** Insights into Land Plant Evolution Garnered from the *Marchantia polymorpha* Genome. *Cell* **171**, 287-304 e215 (2017).
18. Young, N.D. *et al*. The *Medicago* genome provides insight into the evolution of rhizobial symbioses. *Nature* **480**, 520-524 (2011).
19. Tang, H.B. *et al*. An improved genome release (version Mt4.0) for the model legume *Medicago truncatula*. *BMC Genomics* **15**, 312 **(**2014).
20. Worden, A.Z. *et al*. Green evolution and dynamic adaptations revealed by genomes of the marine picoeukaryotes *Micromonas*. *Science* **324**, 268-272 (2009).
21. Ming, R. *et al*. Genome of the long-living sacred lotus (*Nelumbo nucifera* Gaertn.). *Genome Biol*. **14**, R41 (2013).
22. Goff, S.A. *et al*. A draft sequence of the rice genome (*Oryza sativa* L. *ssp. japonica*). *Science* **296**, 92-100 (2002).
23. Ouyang, S. *et al*. The TIGR Rice Genome Annotation Resource: improvements and new features. *Nucleic Acids Res*. **35**, D883-887 (2007).
24. Palenik, B. *et al*. The tiny eukaryote *Ostreococcus* provides genomic insights into the paradox of plankton speciation. *Proc. Natl. Acad. Sci. USA* **104**, 7705-7710 (2007).
25. Lang, D. *et al*. The *P. patens* chromosome-scale assembly reveals moss genome structure and evolution. *Plant J*. **93**, 515-533 (2017).
26. Nystedt, B. *et al*. The Norway spruce genome sequence and conifer genome evolution. *Nature* **497**, 579-584 (2013).

27. Tuskan, G.A. *et al*. The genome of black cottonwood, *Populus trichocarpa* (Torr. & Gray). *Science* **313**, 1596-1604 (2006)
28. International Peach Genome Initiative. The high-quality draft genome of peach (*Prunus persica*) identifies unique patterns of genetic diversity, domestication and genome evolution. *Nat. Genet*. **45**, 487-494 (2013).
29. Banks, J.A. *et al*. The *Selaginella* genome identifies genetic changes associated with the evolution of vascular plants. *Science* **332**, 960-963 (2011).
30. Sato, S. *et al*. The tomato genome sequence provides insights into fleshy fruit evolution. *Nature* **485**, 635-641 (2012).
31. Hirakawa, H. *et al*. Draft genome sequence of eggplant (*Solanum melongena* L.): the representative solanum species indigenous to the old world. *DNA Res*. **21**, 649-660 (2014).
32. Potato Genome Sequencing Consortium. Genome sequence and analysis of the tuber crop potato. *Nature* **475:**189-95 (2011).
33. Paterson, A.H. *et al.* The *Sorghum bicolor* genome and the diversification of grasses. *Nature* **457**, 551-556 (2009).
34. Wang, W. *et al*. The *Spirodela polyrhiza* genome reveals insights into its neotenous reduction fast growth and aquatic lifestyle. *Nat. Commun*. **5**, 3311 (2014).
35. Motamayor, J.C. *et al.* The genome sequence of the most widely cultivated cacao type and its use to identify candidate genes regulating pod color. *Genome Biol.* **14**, r53 **(**2013).
36. Jaillon, O. *et al*. The grapevine genome sequence suggests ancestral hexaploidization in major angiosperm phyla. *Nature* **449**, 463-467 (2007).
37. Prochnik, S.E. *et al*. Genomic analysis of organismal complexity in the multicellular green alga Volvox carteri. *Science* **329**, 223-226 (2010).
38. Schnable, P.S., *et al*. The B73 maize genome: complexity, diversity, and dynamics. *Science* **326**, 1112-1115 (2009).
39. Goodstein, D. M. *et al*. Phytozome: a comparative platform for green plant genomics. *Nucleic Acids Res.* **40**, D1178-1186 (2012).
