# Supplementary material for: Mechanistic insights into the evolution of DUF26-containing proteins in land plants
Source: Commun Biol. 2019 Feb 8;2:56. doi: 10.1038/s42003-019-0306-9 (PMC6368629; doi:10.1038/s42003-019-0306-9)
Supplement: Supplementary file 2 — Description of Additional Supplementary Files [file 42003_2019_306_MOESM2_ESM.pdf]

Description of additional supplementary items:

Supplementary Data 1: Information of DUF26 proteins included in this study. Information of DUF26 protein sequences in study species. Sequences in fasta format are available in Supplementary Data 5.

Supplementary Data 2: The related kinase domains for the CRK kinase domains. PBLAST results for selected CRKs from *Amborella trichopoda*, *Arabidopsis thaliana*, *Oryza sativa* and *Selaginella moellendorffii*. Only amino acid sequence of the kinase domain of each CRK was used as query. Best hit outside the CRKs was marked in the table.

Supplementary Data 3: Gene conversion analyses results.

Supplementary Data 4: Identified orthologs and information of transcriptome data used in analyses.

Supplementary Data 5: Amino acid sequences of DUF26-containing proteins used for phylogenetic analyses provided in fasta format.
