# Supplementary material for: Mechanistic insights into the evolution of DUF26-containing proteins in land plants
Source: Commun Biol. 2019 Feb 8;2:56. doi: 10.1038/s42003-019-0306-9 (PMC6368629; doi:10.1038/s42003-019-0306-9)
Supplement: Supplementary file 3 — Reporting Summary [file 42003_2019_306_MOESM3_ESM.pdf]

nature research

Corresponding author(s): Michael Wrzaczek (michael.wrzaczek@helsinki.fi) Jarkko Salojärvi (jarkko@ntu.edu.sg)

Last updated by author(s): Jan 7, 2019

Reporting Summary

Nature Research wishes to improve the reproducibility of the work that we publish. This form provides structure for consistency and transparency in reporting. For further information on Nature Research policies, see Authors & Referees and the Editorial Policy Checklist.

Statistics

For all statistical analyses, confirm that the following items are present in the figure legend, table legend, main text, or Methods section.

n/a	Confirmed	
☐	☒	The exact sample size (n) for each experimental group/condition, given as a discrete number and unit of measurement
☐	☒	A statement on whether measurements were taken from distinct samples or whether the same sample was measured repeatedly
☐	☒	The statistical test(s) used AND whether they are one- or two-sided<br>*Only common tests should be described solely by name; describe more complex techniques in the Methods section.*
☒	☐	A description of all covariates tested
☐	☒	A description of any assumptions or corrections, such as tests of normality and adjustment for multiple comparisons
☐	☒	A full description of the statistical parameters including central tendency (e.g. means) or other basic estimates (e.g. regression coefficient) AND variation (e.g. standard deviation) or associated estimates of uncertainty (e.g. confidence intervals)
☐	☒	For null hypothesis testing, the test statistic (e.g. F, t, r) with confidence intervals, effect sizes, degrees of freedom and P value noted<br>*Give P values as exact values whenever suitable.*
☒	☐	For Bayesian analysis, information on the choice of priors and Markov chain Monte Carlo settings
☒	☐	For hierarchical and complex designs, identification of the appropriate level for tests and full reporting of outcomes
☒	☐	Estimates of effect sizes (e.g. Cohen's d, Pearson's r), indicating how they were calculated

Our web collection on statistics for biologists contains articles on many of the points above.

Software and code

Policy information about availability of computer code

Data collection	HMMER (version 3.1b2), Wise2 (version 2.4.1), Fgenesh+ (http://www.softberry.com/berry.phtml?topic=fgenes_plus&group=programs&subgroup=gfs), fastq-dump.2 (version 2.5.7), SRA toolkit (version 2.9.0.), ITCrun (TA instruments, version 3.1.10.0), SDSv2.4 (Applied Biosystems).
Data analysis	Guidance (version 2.01), RAxML (version 8.1.3), PASTA, PRANK (version 151120), PAML (version 4.9), R (version 3.4.4), Scipio (version 1.4.1), OrthoMCL (version 2.0.9), Badirate (version 1.35), GenomeTools (version 1.5.4), Synmap (https://genomevolution.org/coge/SynMap.pl), DLCpar (version 1.0.1), FastQC (version 0.11.4), Kallisto (version 0.43.1), Trimmomatic (version 0.36), tximport (version 1.2.0), Wasabi (http://wasabiapp.org/), NanoAnalyze (version 3.5; TA instruments), UCSF Chimera (version 1.12; https://www.cgl.ucsf.edu/chimera/), PyMOL (version 1.8.4.0; https://pymol.org/2/), XDS (version June 2017; http://xds.mpimf-heidelberg.mpg.de/), ShelxD (http://shelx.uni-goettingen.de/shelxd_sm_keywords.php), SHARP (https://www.globalphasing.com/sharp/), Coot (version 0.8.9, https://www2.mrc-lmb.cam.ac.uk/personal/pemsley/coot/), CCP4 (http://www.ccp4.ac.uk/), Refmac5 (http://www.ccp4.ac.uk/html/refmac5.html), Phaser (http://www-structmed.cimr.cam.ac.uk/phaser_obsolete/), Moprobity (http://molprobity.biochem.duke.edu/), GraphPad Prism 6 (version 6.07, GraphPad Software), Geneconv 1.81a (http://www.math.wustl.edu/~sawyer/geneconv/). All R scripts developed to parse the data and visualize the results are available from GitHub.

For manuscripts utilizing custom algorithms or software that are central to the research but not yet described in published literature, software must be made available to editors/reviewers. We strongly encourage code deposition in a community repository (e.g. GitHub). See the Nature Research guidelines for submitting code & software for further information.

Data

Policy information about availability of data

All manuscripts must include a data availability statement. This statement should provide the following information, where applicable:

- Accession codes, unique identifiers, or web links for publicly available datasets
- A list of figures that have associated raw data
- A description of any restrictions on data availability

Detailed phylogenetic trees and alignments used to build them are available through Wasabi.

Field-specific reporting

Please select the one below that is the best fit for your research. If you are not sure, read the appropriate sections before making your selection.

☒ Life sciences ☐ Behavioural & social sciences ☐ Ecological, evolutionary & environmental sciences

For a reference copy of the document with all sections, see nature.com/documents/nr-reporting-summary-flat.pdf

Life sciences study design

All studies must disclose on these points even when the disclosure is negative.

Sample size	Identification and manual curation of 1656 DUF26-containing gene models from high-quality 32 plant and algae genome assemblies. After data exclusions final data set contained 1409 gene models.
Data exclusions	Partial gene models and pseudogenes (all together 247 gene models) were excluded from the analysis.
Replication	1000 bootstraps were ran for each phylogenetic tree to estimate support, additionally results from different datasets were compared to estimate the similarity of the estimated trees.
Randomization	Not applicable
Blinding	Not applicable

Reporting for specific materials, systems and methods

We require information from authors about some types of materials, experimental systems and methods used in many studies. Here, indicate whether each material, system or method listed is relevant to your study. If you are not sure if a list item applies to your research, read the appropriate section before selecting a response.

Materials & experimental systems

n/a	Involved in the study
☒	☐ Antibodies
☐	☒ Eukaryotic cell lines
☒	☐ Palaeontology
☒	☐ Animals and other organisms
☒	☐ Human research participants
☒	☐ Clinical data

Methods

n/a	Involved in the study
☒	☐ ChIP-seq
☒	☐ Flow cytometry
☒	☐ MRI-based neuroimaging

Eukaryotic cell lines

Policy information about cell lines

Cell line source(s)	Insect cell line Tnao38, Hashimoto et al., BMC Biotechnology 2010, doi:10.1186/1472-6750-10-50.
Authentication	The used Tnao38 cell line was not authenticated.
Mycoplasma contamination	The used Tnao38 cell line was not tested for mycoplasma contamination.
Commonly misidentified lines (See ICLAC register)	

The used Tnao38 cell line is listed in the ICLAC database as a commonly misidentified cell line.
The cell line had been originally described as a Ascalapha odorata cell line (Hashimoto et al., BMC Biotechnology 2010, doi:10.1186/1472-6750-10-50.). However, the authors of the original study reported that indeed it is a Trichoplusia ni cell line (Hashimoto et al., BMC Biotechnology 2012, doi:10.1186/1472-6750-12-12).
The cell line is used widespread for secreted protein expression and it is commonly known that it is a Trichoplusia ni cell line.
